# The “sociotype” construct: Gauging the structure and dynamics of human sociality

**DOI:** 10.1371/journal.pone.0189568

**Published:** 2017-12-14

**Authors:** Pedro C. Marijuán, Jesús Montero-Marín, Jorge Navarro, Javier García-Campayo, Raquel del Moral

**Affiliations:** 1 Bioinformation Group, Aragon Institute of Health Science (IACS), Zaragoza, Spain; 2 Bioinformation Group, Aragon Health Research Institute (IIS Aragón), Zaragoza, Spain; 3 Faculty of Health Sciences and Sports, University of Zaragoza, Zaragoza, Spain; 4 Primary Care Prevention and Health Promotion Research Network (RedIAPP), Zaragoza, Spain. Centro de Investigación Biomédica en Red de Salud Mental, CIBERSAM, Spain; University of Exeter, UNITED KINGDOM

## Abstract

Exploring the pertinence of a "sociotype" construct, established along the conceptual chain genotype-phenotype-sociotype, is the essential purpose of the present paper. Further, by following the sociotype’s conceptual guidelines, a new psychometric indicator has been developed in order to gauge the level of social interaction around each individual—the sociotype questionnaire (SOCQ). A first version of this questionnaire has been elaborated by gathering data about the different classes of social bonds (family, friends, acquaintances, and work/study colleagues) in general population and about the dynamic update of these bonds via face-to-face conversation and other modes of interaction. A specific fieldwork was undertaken, involving 1,075 participants, all of them Spanish adults (with diverse social and regional backgrounds). The data obtained were analyzed by means of the correlational method with an analytical cross-sectional design: the number of factors and the consistency and reliability of the resulting scales were evaluated and correlated. The new sociotype indicator resulting from that fieldwork, in spite of its limitations, seems to be valid and reliable, as well as closely associated with widely used metrics of loneliness and psychological distress. It is interesting that the construct noticeably varies throughout the life course and circumstances of individuals, based on their gender and age, and adjusting to the different situations of social networking. This is the first study, to the best of our knowledge, which has tried to reach both a theoretical and an operational formulation of the sociotype construct, by establishing an ad hoc psychometric questionnaire. We think that the information provided by this operational definition opens a new direction of work that could be useful to guide the development and evaluation of programs aimed at improving and strengthening social networking in people at risk, especially for the elderly.

## Introduction

The social nature of our species is one of the few basic consensuses in philosophy and social sciences. As Aristotle wrote in The Politics: “man is by nature a political animal” [1, 2]. However, countless divergent interpretations have been developed thereupon, mostly crystallized around the “nature-nurture” dichotomy. In that complicate scenario, the sociotype construct is close to recent attempts at bridging in between the paleo-anthropological, social networking, and “social physics” studies—so to uncover the social interactions (bonding structures and communication relationships) adaptively demanded by the “social brain” of individuals [1, 3, 4].

In the same way that there is scientific consensus on the validity of the *genotype* and *phenotype* constructs for the human species, notwithstanding their respective degrees of variability, a metrics (or a series of different metrics) could also be developed applying to the relative constancy of the *sociotype*—the social environment and social interactions to which the individuals of our species would be evolutionarily adapted. According to the “social brain hypothesis,” which will be discussed later, the social environment itself has been the major factor in the evolution of our big brains and our enlarged neuro-cognitive capabilities.

The term sociotype has already appeared in the literature, though rather scantily. Seemingly, it was Bogardus [5] who first used it in order to imply the effects of society on the individual’s behavior in a general way, although he did not develop the concept further [6–8]. In psychology, it was put into use by a Jungian oriented school, “socionics”, meaning specific psychological profiles found in well-recognized professions: lawyer, policemen, firefighter, etc. [9]. Initially based on combinations of four psychic functions, the theory has incorporated combinatoric layers of extra complexity upon the sociotypes or socionic types. However, the new use of the term proposed here is closer to the works undertaken by Berry [6] in the biomedical field. He has proposed the sociotype as an integrative term covering internal and external factors for the management of chronic disease, implying the integration of bio-psycho-sociology with systems biology. Independently, some of the present authors have already utilized the term within the triad genotype-phenotype-sociotype, implying the social-evolutionary meaning advocated in the present work [10–13].

From the sociotype perspective, the average brain stimulation coming from relational interactions in the social environment, together with further substitutes and surrogates culturally elaborated, constitutes a mental necessity for the individual’s well-being. Specific fieldwork would be needed, then, in order to appropriately gauge what are the average preferences regarding bonding structures and communication relationships in the different social contexts. Some parallels may be found with recent studies in the biomedical literature [14, 15], in social networks [16–18], and in the “social physics” field [4, 19], though the latter are mostly technological and business oriented. In the present study, which also involves a psychometric perspective, we have attempted the development of an applied tool, a sociotype general questionnaire, tentatively including the main influences and factors related to social intercourse. It is a rather limited–but promising–first step. We have also envisioned a series of future studies and questionnaires covering with more specificity essential features such as age, gender, personality, occupation, culture, etc. Advancing in the sociotype construct by means of further applied tools could provide useful instruments for socioeconomic and communicational analysis, as well as for interventions in psychological and mental healthcare domains—additionally contributing to evidence the noxious consequences of the growing social problem of loneliness.

### Evolutionary roots

Examining the evolutionary roots of the sociotype is necessary for an in-depth comprehension of the new construct. Indeed the crucial novelties of our evolutionary/historical past have revolved around communication matters —e.g. origins of language, emotional expression, group behavior, morals and ethical rules, counting and writing systems, economic and political organizations, knowledge systems, modern media, and so on [20–22]. So fluid and culturally diverse are all the emerging structures of human sociality that, apparently, they defy any precise classification or quantitative specification. However, the presence of a series of significant regularities in the size and structures of social groups, notwithstanding their remarkable variability, suggests the plausibility of a “deep structure” of social bonding for the human species [23–24].

There seems to be an average of social networking, with very ample upper and lower limits, concerning the number and types of bonding relationships that an individual is able to maintain meaningfully. The finding of networking regularities such as the famous “Dunbar’s number” (around 150–200 individual acquaintances) would make evolutionary and anthropological sense [1, 3, 24–27]. These relational findings, integrated within the “social brain hypothesis”, which was originally known as the “Machiavellian intelligence hypothesis” [28], would project an extended clutch on the roots of human sociality, on the origins of language, and on many other traits of social and cultural life. Specifically, the social brain hypothesis has posited that, in primate societies, natural selection has favored larger brains and more complex cognitive capabilities as a means to cope with the challenges of social life [27, 29–32]. Thereafter, due to the overall cortical conformation and brain capacity of our species, vastly enlarged regarding other Anthropoidea, we are able to maintain a really high number of social bonds, meaningfully shared and sustained with the abundant members of our oversized groups. However, like many other brain/mind phenomena, exactly how ‘social bonds’ are made, maintained, differentiated, eroded, restored, finalized, etc. is not sufficiently understood yet—in all probability it is both a universal phenomenon related to crossing some threshold of neuronal complexity and a species-specific phenomenon related to the singularities of the different brains [33].

In the evolution of human societies, language appears as the essential tool for bond-making, although not the only one [29, 34–38]. Human languaging, more often than denotative, becomes cohesive, consensual, identity maker—and above all, a source of mental stimulation. The regular practice of ‘interesting’ conversations induces in our social brain the production of neuropeptides and neurohormones that relieve stress and boost immune system and nervous system’s function [25, 39, 40]. While talking, the specific contents exchanged are often not so important. Rather than the exchange of functional information, it is trivial conversation, gossiping about social acquaintances, what represents the human closest equivalent to grooming exchanges and bond-restoration practices in primate groups [29,36]. Indeed talking may be considered as a new form of grooming in human societies, and comparatively–in energy and physical grounds–it is *virtual*.

### The mental necessity of conversation

Thereafter, the repercussions in human daily life of this new form of virtual grooming can hardly be overstated: talking becomes one of the preferred and most affordable types of mental stimulation. Counting with an appropriate network of people to talk with becomes a necessity for the well-being and mental health of individuals. Having access to, and participating in, amusing conversations becomes an essential ingredient for our social, psychological, and physiological life. The way the different emotions related to social interactions impinge upon language itself and are rearranged within this new channel of expression represents another factor of utmost psychological importance [41–43]. Also, distinguishing several classes of bonds (related to their strength and to their positive or negative valence) turns out to be important in the conversational rewards obtained, as well as in the distinctiveness and degree of surprise of the circulated information, particularly regarding the mentioned preference for the whereabouts of social acquaintances and the importance of the speaker’s own image. The individual fitness within the social group is always at the stake, with relevant gender differences in communication goals and strategies [25, 36, 44, 45].

Within our cognitive dynamics, bonds and conversation are paramount. It can be said both that social bonds claim for their actualization and that linguistic practices claim for their regular realization as well. Both behavioral propensities are reinforcing each other. In fact, the daily conversation/communication budget of each individual has to be apportioned among the different classes of bonds of his/her sociotype so that the talking exercise becomes sufficiently rewarding–provides enough grooming–and that new fitness opportunities may be explored, taking into account the existing diversity of possible encounters and the available communication channels.

The structural and dynamic aspects we are distinguishing in the sociotype (classes of bonds and talking-time budgets) are but two different facets of a unitary social adaptation phenomenon performed by each individual along his/her life. The adaptive sociotype may be conceived as closely following the phenotype’s trajectory along the arch of life—beginnings, development, maturity, and senescence. Analyzing the respective bond structures and conversation-time distributions in these differentiated stages could lead to very interesting comparatives: not only by following the developmental age, but also by taking into account personality, gender, status, professions, cultures, etc.

### The sociotype and the growing problem of loneliness

A number of recent studies on social networks, technologically oriented, have tracked vast amounts of interpersonal exchanges [4, 17, 19, 46], but the metrics of the relational structures necessary for personal well-being and mental health have hardly been addressed yet. Hopefully, the progressive delineation of a sociotype construct, susceptible of both theoretical and empirical demarcation, might contribute to a better understanding of the structures and dynamics of human sociality, and might provide some practical help when sociality itself is in crisis, as seems to be happening with the current “epidemics of loneliness” affecting large population tracts and particularly the elderly [47–53].

At a social/economic scale, the diminished relationships and bonding structures of “social capital” would penalize the activities of daily life and would decrease the individual’s well-being [17, 48, 54]. The evidence in fast-developing countries is that economic growth and technological development spurred by the ‘information revolution’ have gone hand-in-hand with an increase in behavioral disorders, family disintegration, social exclusion, and lower social trust [55, 56]. In the 1985 US Census, the average number of confidants was three; in the 2004 Census the average was 2, but the most common figure was zero confidants for almost 25% [57]. The phenomenon is similar in most Western countries, where the ubiquitous presence of Media and of information and communication technologies (ICTs) has dramatically altered life styles. In recent years, there has been a significant transfer of social life and collective entertainment activities to individualized activities such as computer games, Internet, TV watching, and the new online social networks. However, it is unclear the effect that such ICTs pervasiveness and overuse are having in our social relationships and quality of life. In what extent could computers, cell phones, and TVs replace our need of face-to-face relationships? [45, 49, 58–61]. The balance between positive and negative factors is not settled yet. It is at least significant that depression and suicide rates have increased dramatically in the last three decades; and that mental disorders nowadays represent a global disease burden that surpasses cardiovascular diseases and cancer [62–64].

From a psychological and biomedical perspective, in spite of the pervasive loneliness and lack of meaningful relationships in contemporary societies, there is a dearth of adequate indicators gauging the conversational activities of the individual. Actually, none of the existing questionnaires on related topics (e.g. UCLA loneliness scale, MSPSS, SNI, Duke, SELSA, MOS, SSB, Zimet, de Jong, etc.) seems to be centered in the basic, face-to-face relational phenomena focused by the sociotype. Precisely, this is the kind of information that a few of the most recent enquiries are beginning to ask for [65, 66].

### The present study

Subsequently, the empirical part of the present study has tried to develop a new psychometric indicator related to the social interactions ‘adaptively’ demanded by the social brain of each individual. By means of a specific survey, different kinds of psychosocial data were gathered around the structure of individuals’ social bonds and on their dynamic update via conversation. According to criteria frequently used in other studies [most important sources have been: 4, 29, 47, 48, 57, 67, 68], we distinguished between three main relational scenarios: the own residence, public spaces, and the workplace (or study). We also distinguished between four types of relationships: family, close friends, acquaintances, and work/study colleagues [in contraposition to the three levels in 67]. This division has oriented the basic structure of the new psychometric indicator. Essentially, the present study–and its related fieldwork–has aimed to develop an accurate and applicable operationalization of a sociotype-inspired psychometric indicator, evaluating its structure, internal consistence, and convergent validity.

We have also aimed to assess the sociotype’s explanatory power to account for loneliness and psychological distress, throughout the relationships with UCLA and GHQ-12 measures, both considered as individual’s well-being metrics likely influenced by the sociotype outcomes; and additionally we have explored possible differences due to age, personality, and gender. Overall, the following working hypotheses have been considered:

1. The sociotype can be measured with appropriate psychometric features as a general unique dimension, and also by means of specific sub-domains; the statistical analysis has to show both a confirmative factor structure and a robust internal consistency for the total scale and for the sub-domains.
2. The sociotype scale should converge with the other constructs related to psychological states of loneliness and distress (UCLA, GHQ-12), demonstrating its validity and relevance by means of a high, significant correlation.
3. The sociotype scale should correlate with the different dimensions of personality, positively with extraversion and negatively with neuroticism and psychoticism (the lie dimension could be more complex, depending on the different domains).
4. The sociotype scale would change across the successive stages of life, with different subscales increasing/decreasing their levels throughout the life course, and expecting an overall decline for the elderly.
5. According to gender, the sociotype sub-scales could show significant differences: traditionally, a stronger social network for males in terms of work/study colleagues would be expected, while for females the stronger networking should appear in terms of family.

Given that the general human need to connect–to which the sociotype refers–seems to be a universal adaptive trait [29], an open exploratory question would concern the relative constancy of the overall sociotype measure. It could be hypothesized as being caught in a similar range of variability for most individuals, although strongly biased in its constitutive sub-domains by gender and age, and by different personal, social and cultural factors and influences—see for instance the “epidemics of loneliness” that affects more intensely the elderly in modern societies. The sociotype means the endless adaptation to the different possibilities of social intercourse around the individual.

Shedding light on all these structural differences and social influences would permit a better characterization of the structure and dynamics of human sociality, guiding the implementation of public programs aimed at strengthening social networking.

## Material and methods

### Study design

The structure of the study was based on the correlational method with an analytical cross-sectional design. The whole measurements were obtained by means of the self-assessment technique, using a set of questionnaires via Internet, complemented with face-to-face interviews as well.

### Participants

Most of the participants accessed the survey via Internet, but around 15% were face-to-face interviews (for the elderly), intentionally trying to cover for the different gender, age, and status characteristics. In all cases, the inclusion criteria were age >18 years, being able to read and write Spanish, and not suffering from severe physical or mental disorders. The final number of participants (n = 1,075) exceeded the validity evaluation criterion [69], resulting in a sample that was psychometrically adequate for the study. The main characteristics of the sample are presented in Section 3.

### Procedure and ethics statement

In order to hypothesize the structure of the sociotype model, we started with a qualitative study looking for the main characteristics of social networking and conversational habits in different ages, by means of semi-structured interviews (involving narratives of personal experiences, moods, appreciation of new communication technologies, etc.). See (S2 and S3 Files). This qualitative study involved 45 interviews (conveniently diversified by age, gender and class) [70]. A total of 45 participants were recruited (26 female and 19 male), with a mean age of 60.4 (SD = 22.26); they were intentionally selected to try to cover the referred socio-demographic characteristics. All of these participants signed an informed-consent form approved by the Ethical Committee of Aragón, Spain (CEICA). The interviews were recorded and transcribed by the interviewer herself (Raquel del Moral). These records were subject to content analysis by three researchers (Raquel del Moral, Pedro C. Marijuán, and Jesús Montero-Marín), who independently identified the emerging categories according to which the generality of the topics could be encoded [71]. The broad frameworks of the possible sociotype interactions were tentatively identified. Next, we attempted to determine which conceptual aspects of the sociotype were typical of these frameworks. We empirically defined each of the emerging categories by discussing their ability to capture the different interactions. The appropriate adjustments were made by consensus to ensure that each definition would be comprehensive and exclusive of the others [72].

That initial qualitative experience provided a number of data about how the theoretical notion of the sociotype was felt in different personal circumstances and social domains. Subsequently, a preliminary pilot fieldwork was developed applying a convenience sampling to 165 subjects, all of them students from two education centers, which were interrogated using a very preliminary draft-model [12]. As a result of these works, and in consonance with the above cited literature, we proposed the emergent dimensions of ‘family’, ‘friends’, ‘acquaintances’, and where applicable ‘study or work mates’, as the main factors that a sociotype basic definition should include. Further, a series of 32 selected questions were proposed to conform the initial version of the “Sociotype Questionnaire” (SOCQ), which will be described below in its developmental process and final version.

The duration of the complete survey was approximately half an hour. Each of the participants was presented with an initial description of the survey (with an informed consent form), which introduced the aims of the study, the advantages/disadvantages of participating, and notification that the data would be processed anonymously (S4 File). An online platform gave support to the completion of the survey and data collection (http://www.surveymonkey.com). A research psychologist administered the questionnaires performed in the face-to-face format (for most elderly participants); afterwards the collected data were dumped on the online platform (S1 Table).

The Ethical Committee of Aragón (CEICA) had previously approved this study (Act: CP13/2014). All the participants provided their informed consent before completing the survey, either by reading the project information and providing verbal consent (face-to-face format), or by explicitly accepting the study conditions (online platform). Given the procedure followed and the kind of generic data requested, the anonymity of the participants in the survey was granted.

### Measurements

- Background variables: The survey recorded a set of socio-demographic variables providing a general view of the social circumstances of participants, such as: sex, age, relationships (‘with partner/married’, ‘single’, ‘separate/divorced’, ‘widow/widower’), connivance (‘alone’, ‘partner’, ‘partner and children’, ‘other family’, ‘friends’, ‘residence’), place (‘rural’, ‘urban’), education (‘no studies’, ‘primary’, ‘high school’, ‘university’), employment (‘student’, ‘unemployed’, ‘employed’, ‘retired’), salary (‘<Minimum Wage’, ‘1–2 MW’, ‘2–4 MW’, ‘>4 MW’), social satisfaction (using a Visual Analogical Scale–VAS, from 0 to 100).
- Sociotype Questionnaire (SOCQ): Subjects were asked a set of 32 items, assessing the quality of their relationships with ‘family’, ‘friends’, ‘acquaintances’, and ‘study/work’ mates (8 questions for each one). The first three domains were proposed as subscales of a general sociotype factor, and the fourth one was proposed as an independent scale, to be used when applicable (in this case, 49,5% employed and 11,3% students). The items were developed by a multidisciplinary expert panel (including biologists, psychologists and sociologists), who included the main characteristics of each domain by consensus. The wording of the items was guided by a table of content specifications, enabling their fit, conceptual validity, and representativeness. The number of items was over-dimensioned to select those with the best psychometric properties. In order to counteract the effects of response styles and biases, the survey utilized a forced-choice response format, rating the degree of agreement with each of the statements, some of them in reverse score, using a Likert-type scale with 6 response options, from 0 (never) to 5 (always). See (S1 File).
- General Health Questionnaire (GHQ-12): This is the most extensively used screening instrument to measure psychological distress, being attractive because of its brevity (12 items) [73]. Its psychometric properties have been studied in several countries [74], applying to various types of population, e.g., elderly [75] and urological patients [76]. We used the Spanish validated version [77, 78], with α = 0.76. The correction was conducted assigning values from 0 to 3 to the different possible answers (S5 File).
- The Revised UCLA Loneliness Scale (RULS): This widely used questionnaire consists in a one-dimensional 20-item scale, designed to measure subjective feelings of loneliness and social isolation [79]. It is a revised version of the original UCLA Loneliness Scale [80]. The Spanish validated version herein used counts with adequate psychometric properties, α = 0.94 [81]. Participants rate each item on a Likert-type scale ranged from 1 (never) to 4 (often). See (S6 File).
- Eysenck Personality Questionnaire-Revised (EPQ-R): This questionnaire measures three major dimensions that account for most of the variance in personality [82]. The EPQ-R is an excellent choice to represent the personality domain. This measure has proven useful for numerous applications in human resources, career counseling, clinical settings and biomedical research. A validated Spanish version of this questionnaire was used, with adequate psychometric properties [83]. The EPQ-R scales are: ‘extraversion’ (α = 0.82), ‘neuroticism’ (α = 0.86), ‘psychoticism’ (α = 0.73), and ‘lie’ (α = 0.76). The total number of items forming the Spanish version of EPQ-R is 83, and they are answered assigning ‘yes’ or ‘not’ (S7 File).

### Statistical analysis

Means (SD) and frequencies (percentages) were calculated on the socio-demographic data. From the proposed items of the sociotype questionnaire, we selected those with the best discrimination coefficient (item-rest coefficient) in their respective domain, taking into account the criterion of ≥0.30 from the Classic Test Theory point of view [84]. To analyze the factor structure, we randomly split the sample into two halves: the first sub-sample (n1 = 538) for the initial Exploratory Factor Analysis (EFA), and the second sub-sample (n2 = 537) for the Confirmatory Factor Analysis (CFA). Mardia’s coefficients [85] were estimated to evaluate items’ distribution in multivariate terms. Polychoric correlation matrices, especially developed for the analysis of relationships between polytomous categorical variables, were calculated; KMO index as a measure of sampling adequacy, and Barlett’s test of sphericity to check if there is enough redundancy between the items to be summarized with a few number of factors, were verified [86], ensuring beforehand that the determinant of the matrices were greater than 0.00001 in order to discard possible problems of multi-collinearity [87].

We used parallel analysis [88] to identify the number of factors, replacing the raw data by optimal implementation based on minimum rank factor analysis after generating 500 random correlation matrices [89]. A factor is significant if the associated eigenvalue is bigger than that corresponding to a 95th percentile of the eigenvalues derived from the random dataset. This method is the best solution to decide the number-of-factors-to-retain [90, 91]). The unweighted least squares (ULS) was the method used for factor extraction in the EFA, in view of its demonstrated robustness, especially when working with polychoric matrices [92]. The rotation method was Promax (k = 4.00), given the correlated solution expected, using raw varimax as clever rotation start. To select the items to be included in each factor, we used the criterion of loadings w >0.5 [93], and we used the Item Response Theory (IRT) parameterization by the multidimensional normal-ogive graded response model, which is derived from the assumption of normally distributed measurement error [94], with an ≥0.65 as criterion to interpret the pattern of item discriminations. The percentage of explained variance in each item by means of communality values (h2) was calculated. We tested the appropriateness of fit by using the goodness of fit index (GFI) and the root mean square of standardized residuals (RMSR), which are explained bellow. From the proposed items, we selected those with the best discrimination coefficient in their respective domain, taking into account the Classic Test Theory point of view [84], and using the criterion of item-rest correlations ≥0.30 in the corresponding domain.

We examined the absolute and incremental fit of the emergent SOCQ model by confirmatory factor analysis, applying unweighted least squares, and using the GFI, the adjusted goodness-of-fit index (AGFI), the root mean square of standardized residuals (RMSR), the normed-fit-index (NFI), and Bollen’s relative-fit-index (RFI). GFI and AGFI refer to explained variance and values >0.90 are acceptable [95]. RMSR is the standardized difference between the observed and the predicted covariance, indicating good fit values <0.08 [96]. NFI measures the proportional reduction in the adjustment function when going from null to the proposed model and is considered acceptable when >0.90 [97]. RFI takes into account the discrepancy for the model evaluated and for the baseline model and it is very good close to 1 [98]. Standardized factor saturations (λ and γ), from an analytical point of view of the models, were also considered.

We examined the internal consistency of the scales using congeneric, tau-equivalent and parallel models of reliability [99]. The congeneric model is the least restrictive and assumes that each individual item measures the same latent variable, with possibly different scales, degrees of precision, and magnitude of error. The tau-equivalent model implies that individual items measure the same latent variable on the same scale and with the same degree of precision, but with possibly different degrees of error. The parallel model is the most restrictive model; it assumes that all items must measure the same latent variable on the same scale, with the same degree of precision and with the same amount of error. In order to reach parsimony, we chose the more restrictive model that fit good enough with the data, applying the ULS method [100]. The reliability value was calculated by squaring the implied correlation between the composite latent true variable and the composite observed variable, to arrive at the percentage of the total observed variance that was accounted for by the true variable [100]. Item-rest and mean item-rest correlations were also calculated to assess the degree of relationship among the finally selected items.

We used the SOCQ dimensions as independent variables in multivariate linear regression models, in order to assess the contribution of the sociotype construct to explain ‘loneliness’ and ‘psychological distress’, controlling the possible influence of the personality traits. Previously, we evaluated the degree of association regarding all the constructs, by means of Spearman’s R coefficients. Standardized beta coefficients were used to assess the individual contribution of each variable, and the Wald test was used to evaluate their significance. Adjusted multiple determination coefficients (R2y.123) were also calculated to observe their grouped explanatory power, and their significance was assessed by means of analysis of variance [101]. Partial correlation coefficients (Ry3.12) −to indicate the correlation between two variables when the effect of the other variables included in the equation are removed− and semi-partial correlation coefficients (Ry(3.12)) −the square of which shows the increase in the coefficient of determination after including a specific variable in a model, partialising the influence of the other included variables− were also calculated. The basic assumptions of the regression models were evaluated by using the K-S test over the conditional distribution of the residuals to ensure they were normally distributed, by the Durbin-Watson test to rule out possible autocorrelations in the error terms (adequate with a roughly value = 2.00), and by the tolerance values (1- the squared multiple correlation of a given regressor with the remaining), to discard co-linearity problems [101].

Student's t-test for independent measurements were used to contrast possible differences in the SOCQ dimensions by sex, and the one-way ANOVA in the case of age (groups: ‘18–30 years’, ‘31–45 years’, ‘46–65 years’ and ‘>65 years’). The basic assumptions of both contrasts (independence, normality and heterocedasticity) were revised. All the tests used were bilateral (α<0.05). Packages SPSS v19, FACTOR v10, and AMOS v20 were used to conduct the statistical analysis.

## Results

### Socio-demographic characteristics of participants

A total of 1,075 participants completed the study. All of them were Spanish (with diverse regional backgrounds), 66.8% females and 33.2% males, between the ages of 18–95 years (Mean = 49.79; SD = 21.47), 52.3% of them with partner or married and 25.6% singles, 87.8% living in an urban context, 58% with university studies, 49.7% employed, and 28.1% retired. The main socio-demographic characteristics of all the participants are shown in Table 1.

**Table 1:** Characteristics of the study participants (n = 1,075).

*Sex*, females (%)	718 (66.8)
*Age*, Md (SD)	49.79 (21.47)
*Stable relationship* (%)	
with partner/married	562 (52.3)
single	275 (25.6)
separate/divorced	63 (5.8)
widow/widower	175 (16.3)
*Connivance* (%)	
Alone	264 (24.6)
partner	290 (27.0)
partner and children	255 (23.8)
other family	170 (15.8)
friends	60 (5.5)
residence	36 (3.3)
*Place* (%)	
Rural	131 (12.2)
Urban	944 (87.8)
*Education* (%)	
no studies	157 (14.6)
primary	151 (14.0)
high school	142 (13.2)
university	625 (58.2)
*Employment* (%)	
student	122 (11.3)
unemployed	117 (10.9)
employed	534 (49.7)
retired	302 (28.1)
*Salary* (%)	
<Minimum wage (MW)	256 (23.8)
1–2 MW	389 (36.2)
2–4 MW	332 (30.9)
>4 MW	98 (9.1)
*Social satisfaction (VAS 0–100)*, Md (SD)	72.52 (21.35)

Md = Mean; SD = Standard Deviation; Number and percentage (%). MW = 650€

### Exploratory Factor Analysis (EFA)

Table 2 shows the features of the 16 general SOCQ items finally selected according to the method previously described based on the Classical Theory of Tests (n_1_ = 538) [Mardia’s = 37.66 (p<0.001); KMO = 0.80; Bartlett's = 2,927.40 (p<0.001)]. Parallel analysis identified three factors, explaining 69.8% of the variance. F_1_ presented topics associated with ‘friends’, F_2_ with ‘family’, and F_3_ with ‘acquaintances’. The Schmid-Leiman second order factor solution presented the values of F_1_ = 0.55, F_2_ = 0.67 and F_3_ = 0.54. The model presented appropriate fit (GFI = 0.99; RMSR = 0.04). Table 2 also shows the characteristics of the SOCQ at work/studies items (n = 328) [Mardia’s = 9.24 (p<0.001); KMO = 0.82; Bartlett's = 350.70 (p<0.001)]. Parallel analysis identified one factor, explaining 69.9% of the variance. F_1_ presented topics related to ‘mates at work/studies’. The model presented appropriate fit (GFI = 1.00; RMSR = 0.03).

**Table 2:** Psychometric features of the SOCQ by using Exploratory Factor Analysis*.

**General SOCQ**	**Mn**	**SD**	**a_1_**	**a_2_**	**a_3_**	**h^2^**	**w_1_**	**w_2_**	**w_3_**
**Friends**									
5. I speak and relate with my friends	3.44	1.48	2.06	-0.14	0.21	0.81	**0.89**	-0.06	0.09
6. I have friends to tell and share problems	3.45	1.65	2.24	-0.17	0.13	0.83	**0.92**	-0.07	0.06
7. I consider important to maintain relationships with friends	4.14	1.39	2.09	-0.03	0.02	0.81	**0.90**	-0.01	0.01
8. I have fun and laugh with my friends	3.59	1.41	1.46	0.16	-0.20	0.68	**0.82**	0.09	-0.11
**Family**									
1. I speak and relate with my family	4.39	0.97	-1.37	2.12	0.04	0.81	-0.16	**0.94**	0.02
2. My family is important for me	4.74	0.76	-0.30	2.24	0.23	0.83	-0.12	**0.91**	0.10
3. The family members care about me	4.49	1.00	-0.06	1.35	-0.01	0.64	-0.04	**0.81**	-0.01
4. I have fun and laugh with my family	3.65	1.20	0.35	0.73	-0.16	0.43	0.26	**0.55**	-0.12
**Acquaintances**									
9. I speak and relate comfortably with acquaintances	3.61	1.19	0.08	0.16	0.84	0.47	0.06	0.12	**0.61**
10. It costs me make conversation with people I do not know (r)	3.19	1.33	-0.02	-0.10	0.75	0.34	-0.01	-0.08	**0.61**
11. It is easy for me to win support from acquaintances	2.29	1.48	0.08	-0.10	0.66	0.24	0.08	-0.09	**0.52**
12. Relations with my acquaintances are forced (r)	3.53	1.05	-0.03	0.06	0.82	0.42	-0.02	0.05	**0.63**
% of variance (real-data)							38.70	18.80	13.90
% of variance (95% percentile of random)							22.10	16.90	13.10
**Sociotype at work/studies**					**Mn**	**SD**	**a**_**1**_	**h**^**2**^	**w**_**1**_
13. I speak and relate satisfactorily with my peers					3.87	1.17	1.25	0.61	**0.78**
14. I have personal trust in my peers					3.34	1.31	1.32	0.64	**0.80**
15. When talking with peers they take me into account					3.48	1.27	1.31	0.63	**0.79**
16. I feel valued by my peers					3.45	1.20	1.04	0.52	**0.72**
% of variance (real-data)									82.5
% of variance (95% percentile of random)									66.9

*SOCQ exploratory measurement model from sub-sample 1 (general sociotype n_1_ = 538; sociotype at work/studies n = 328). Mn = mean. SD = standard deviation. w_1_, w_2_ & w_3_ = weights on the first-order factors. h^2^ = communality. a_1_, a_2_ & a_3_ = IRT discrimination. r = reverse score.

### Confirmatory Factor Analysis (CFA)

**Fig 1:**
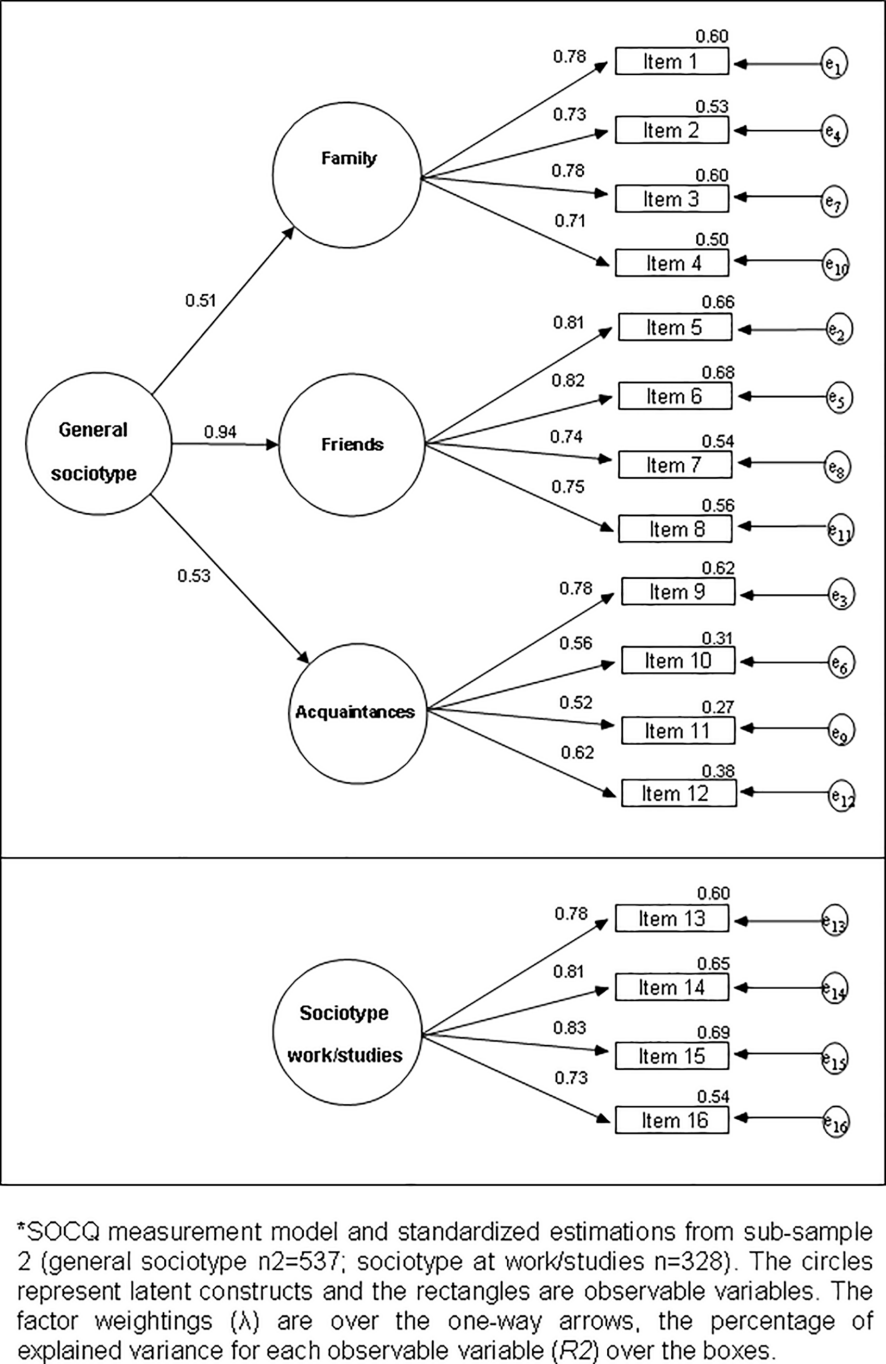
Analytical perspective of the SOCQ by using confirmatory factor analysis.

The characteristics of the general SOCQ matrix (n_2_ = 537), were: Mardia’s = 33.28 (p<0.001); KMO = 0.84; Bartlett's = 3,140.90 (p<0.001). Fig 1 shows the general SOCQ structure using CFA from an analytical and standardized point of view. The three first order factors turned out to be highly influenced by a general second order factor (G), with loadings over F_1_ = 0.51, F_2_ = 0.94, and F_3_ = 0.53, and explaining 73.7% of the variance. The item loadings with regard to their respective latent factor were high (from 0.52 to 0.82). The general SOCQ structure presented adequate fit indices with no using correlations between the error terms (GFI = 0.99; RSMR = 0.05; AGFI = 0.98; NFI = 0.98; RFI = 0.97). The characteristics of the SOCQ at work or studies matrix (n = 328) were: Mardia’s = 11.29 (p<0.001); KMO = 0.82; Bartlett's = 1,003.78 (p<0.001). Fig 1 also shows the SOCQ at work/studies structure by using CFA. The only one factor explained 71.7% of the variance, with loadings from 0.73 to 0.83. The SOCQ at work/studies structure presented adequate fit with no using correlations between the error terms (GFI = 0.99; RSMR = 0.02; AGFI = 0.99; NFI = 0.99; RFI = 0.99).

### Reliability models of the SOCQ

Table 3 shows the reliability models tested for the SOCQ. All the scales and sub-scales fitted better with the congeneric model, being the estimates obtained of R = 0.81 for the ‘general SOCQ’, R = 0.81 for ‘family’, R = 0.90 for ‘friends’, R = 0.71 for ‘acquaintances’, R = 0.87 for ‘work/studies’. The average of item-rest values for the ‘general SOCQ’ was 0.52, being of 0.64 for the ‘family’ sub-scale, of 0.77 for the ‘friends’ sub-scale, of 0.45 for the ‘acquaintances’ sub-scale, and of 0.72 for the ‘work/studies’ sub-scale.

**Table 3:** Fix indices for the reliability models of the SOCQ.

Scales/Factors	R	CMIN	NPAR	GFI	AGFI	RSMR	NFI	RFI
*General SOCQ*		108.89	27	0.99	0.98	0.05	0.98	0.97
Congeneric	0.81	2,081.98	24	0.93	0.91	0.15	0.88	0.85
Tau-equivalent	0.80	7,771.96	13	0.75	0.71	0.19	0.54	0.53
Parallel	0.80	9,422.95	2	0.70	0.69	0.18	0.44	0.52
Family								
Congeneric	0.81	0.55	8	0.99	0.99	0.01	0.99	0.99
Tau-equivalent	0.81	23.91	5	0.99	0.99	0.05	0.99	0.99
Parallel	0.81	168.77	2	0.95	0.94	0.06	0.90	0.92
Friends								
Congeneric	0.90	11.16	8	0.99	0.99	0.02	0.99	0.99
Tau-equivalent	0.90	268.36	5	0.99	0.97	0.08	0.98	0.97
Parallel	0.90	482.94	2	0.97	0.97	0.04	0.96	0.97
Acquaintances								
Congeneric	0.71	7.19	8	0.99	0.99	0.02	0.99	0.98
Tau-equivalent	0.70	52.71	5	0.99	0.98	0.05	0.95	0.95
Parallel	0.70	362.65	2	0.94	0.93	0.08	0.68	0.76
*Sociotype at work/studies*		4.12	8	0.99	0.99	0.02	0.99	0.99
Congeneric	0.87	5.36	8	0.99	0.99	0.02	0.99	0.99
Tau-equivalent	0.87	18.38	5	0.99	0.99	0.03	0.99	0.99
Parallel	0.87	30.27	2	0.99	0.99	0.04	0.99	0.99

R = Reliability; CMIN = mínimum value of the discrepancy; NPAR = number of parameters being estimated; GFI = Goodness of Fit Index; RSMR = Root Mean Square of the Standardized Residuals; AGFI = Adjusted Goodness of Fit Index; NFI = Normed Fit Index; RFI = Relative Fit Index.

### Explanatory power of the SOCQ regarding loneliness and psychological distress

The SOCQ factors showed important associations with the other constructs (Table 4).

**Table 4:** Relationships of the SOCQ dimensions with the other constructs.

	Mn	SD	1	2	3	4	5	6	7	8	9	10
**1. General SOCQ** (0–60)	44.82	8.27										
**2. Work/studies** (0–20)	13.87	3.93	0.50*									
**3. Family** (0–20)	17.28	3.16	0.65*	0.37*								
**4. Friends** (0–20)	14.98	4.94	0.82*	0.46*	0.33*							
**5. Acquaintances** (0–20)	12.55	3.39	0.64*	0.31*	0.19*	0.26*						
**6. Loneliness** (20–80)	34.74	10.68	-0.71*	-0.48*	-0.49*	-0.56*	-0.46*					
**7. Psychological distress** (0–36)	12.14	6.05	-0.42*	-0.29*	-0.26*	-0.34*	-0.29*	0.52*				
**8. Extraversion** (0–19)	11.91	4.50	0.62*	0.29*	0.23*	0.57*	0.44*	-0.54*	-0.38*			
**9. Neuroticism** (0–23)	9.91	5.54	-0.33*	-0.25*	-0.23*	-0.23*	-0.26*	0.51*	0.57*	-0.30*		
**10. Psychoticism** (0–23)	4.41	3.04	-0.25*	-0.22*	-0.30*	-0.16*	-0.10*	0.26*	0.17*	-0.09^**‡**^	0.21*	
**11. Lie** (0–18)	10.60	4.10	-0.05	-0.03	0.03	-0.24*	0.19*	-0.02	0.05	-0.21*	-0.01	-0.06

Mn: mean; SD: standard deviation. The rest of values are Spearman’s correlations.

* p<0.001.

**‡** p<0.01. Possible range in brackets

The explanatory power of the regression models was very high (Table 5). ‘Loneliness’ was explained (R^2^ = 0.62; p<0.001) by ‘family’ (Beta = -0.24; p<0.001), ‘friends’ (Beta = -0.29; p<0.001), ‘acquaintances’ (Beta = -0.18; p<0.001), ‘extraversion’ (Beta = -0.17; p<0.001), ‘neuroticism’ (Beta = 0.29; p<0.001) and ‘psychoticism’ (Beta = 0.05; p = 0.040). ‘Psychological distress’ was explained (R^2^ = 0.42; p<0.001) by ‘family’ (Beta = -0.08; p = 0.005), ‘friends’ (Beta = -0.09; p = 0.009), ‘acquaintances’ (Beta = -0.08; p = 0.010), ‘work/studies’ (Beta = -0.15; p<0.001), ‘extraversion’ (Beta = -0.10; p = 0.005), ‘neuroticism’ (Beta = 0.45; p<0.001). It was possible to accept the basic assumptions needed to go ahead with the regression, with tolerance values from 0.55 to 0.85.

**Table 5:** Regression models for the SOCQ with regard to loneliness and psychological distress.

**Loneliness**	**R**_**y.123**_	**R**^**2**^_**y.123**_	**F (df**_**1**_ **/ df**_**2**_**)** **p**^**a**^	**Se**	**DW**	**p**^**b**^
	0.78	0.62	176.21 (8 / 907) <0.001	6.72	1.88	0.217
	**R**_**y3.12**_	**R**_**y(3.12)**_	**B (95% CI)**	**Se**	**Beta**	**p**^**c**^
*Intercept*			65.40 (61.52–69.28)	1.98		<0.001
family	-0.33	-0.22	-0.83 (-0.99 –-0.68)	0.08	-0.24	<0.001
friends	-0.34	-0.22	-0.63 (-0.75 –-0.52)	0.06	-0.29	<0.001
acquaintances	-0.22	-0.14	-0.54 (-0.69 –-0.38)	0.08	-0.18	<0.001
work/studies	0.03	0.02	0.04 (-0.04–0.12)	0.04	0.03	0.328
Extraversion	-0.20	-0.13	-0.41 (-0.54 –-0.28)	0.07	-0.17	<0.001
Neuroticism	0.39	0.27	0.56 (0.48–0.65)	0.04	0.29	<0.001
Psychoticism	0.07	0.04	0.16 (0.01–0.32)	0.08	0.05	0.040
Lie	-0.09	-0.06	-0.17 (-0.30 –-0.05)	0.07	-0.07	0.008
**Psychological distress**	**R**_**y.123**_	**R**^**2**^_**y.123**_	**F (df**_**1**_ **/ df**_**2**_**)** **p**^**a**^	**Se**	**DW**	**p**^**b**^
	0.64	0.42	80.16 (8 / 907) <0.001	4.67	1.95	0.137
	**R**_**y3.12**_	**R**_**y(3.12)**_	**B (95% CI)**	**Se**	**Beta**	**p**^**c**^
*Intercept*			17.00 (14.30–10.69)	1.37		<0.001
family	-0.09	-0.07	-0.15 (-0.26 –-0.05)	0.06	-0.08	0.005
friends	-0.09	-0.07	-0.11 (-0.19 –-0.03)	0.04	-0.09	0.009
acquaintances	-0.09	-0.07	-0.14 (-0.24 –-0.03)	0.05	-0.08	0.010
work/studies	-0.15	-0.12	-0.13 (-0.18 –-0.07)	0.03	-0.15	<0.001
Extraversion	-0.09	-0.07	-0.13 (-0.22 –-0.04)	0.05	-0.10	0.005
Neuroticism	0.48	0.41	0.50 (0.44–0.56)	0.03	0.45	<0.001
Psychoticism	-0.02	-0.02	-0.03 (-0.14–0.08)	0.06	-0.02	0.552
Lie	-0.05	-0.04	-0.07 (-0.16–0.02)	0.05	-0.05	0.136

R_y.123_ = multiple correlation coefficient. R^2^_y.123_ = coefficient of multiple determination.

p^a^ = p value for variance analysis associated with the regression. Se = standard error. DW = Dubin-Watson value.

p^b^ = p value for K-S test for normality contrast on residuals. R_y3.12_ = partial correlation coefficient. R_y(3.12)_ = semi-partial correlation coefficient. B = regression slope. CI = confidence interval. Beta = standardised slope.

p^c^ = p value of Wald test result.

### Differences in SOCQ according to sex and age

As we can see (Table 6), males presented higher scores in ‘work/studies’ than females [(Mn = 10.28; SD = 6.90) vs. (Mn = 8.99; SD = 7.35); p = 0.005]; while females did it in ‘family’ [(Mn = 17.01; SD = 3.18) vs. (Mn = 17.46; SD = 3.10); p = 0.027]. In terms of age, the SOCQ-general, ‘friends’ and ‘work/studies’ showed a decreasing trend (p<0.001), ‘acquaintances’ showed an increasing trend (p<0.001), and ‘family’ did not show differences among age groups (p = 0.333). ‘Social satisfaction’, measured by the VAS, did not show significant changes either by sex (p = 0.217) or by age (p = 0.262). The general assumptions of independent groups to develop the analyses were fulfilled.

**Table 6:** SOCQ differences according to sex and age.

	SEX			AGE				
Variable (range)	male (n = 357)	female (n = 718)	p^a^	18–30 years (n = 241)	31–45 years (n = 290)	46–65 years (n = 256)	>65 years (n = 288)	p^b^
**SOCQ-general** (0–60) Mn (SD)	44.48 (8.27)	44.97 (8.27)	0.825	46.33 (6.96)	45.54 (6.75)	44.70 (8.61)	43.04 (9.78)	<0.001
**Family** (0–20) Mn (SD)	17.01 (3.18)	17.46 (3.10)	0.027	17.33 (3.04)	17.31 (2.79)	16.92 (3.80)	17.38 (2.94)	0.333
**Friends** (0–20) Mn (SD)	14.90 (4.79)	15.14 (4.94)	0.454	17.16 (3.04)	15.99 (3.53)	15.09 (4.06)	12.50 (6.50)	<0.001
**Acquaintances** (0–20) Mn (SD)	12.60 (3.31)	12.53 (3.42)	0.778	11.77 (3.40)	12.16 (3.15)	12.78 (2.91)	13.29 (3.75)	<0.001
**Work/studies** (0–20) Mn (SD)	10.28 (6.90)	8.99 (7.35)	0.005	13.39 (5.07)	12.09 (5.78)	11.31 (6.12)	1.72 (4.87)	<0.001
**Social satisfaction** (VAS 0–100) Mn (SD)	73.75 (20.01)	71.95 (21.93)	0.217	73.40 (19.16)	74.14 (19.63)	70.38 (21.69)	71.98 (24.23)	0.262

^a^ t-contrast for independent groups.

^b^ one-way analysis of variance (ANOVA).

## Discussion

This is the first study, to the best of our knowledge, that has tried to reach both a theoretical and an operational approach to the sociotype construct, as portraying and delimiting the fundamental structure of social relationships of a person [11–13]. The relative constancy of a compound of relational layers and their associated dynamics of actualization would accompany each individual along the advancement of his/her life cycle. Given the orientation herein followed toward the use of this new indicator in areas of mental health and general well-being, the other accompanying questionnaires are related to loneliness, psychological distress, and personality dimensions; they have contributed to delimit and establish the horizon of this first applied exploration.

We have found that the proposed psychometric indicator was valid in terms of structure and reliable enough in terms of internal consistency. A general scale was established consisting of the subscales family, friends, acquaintances, and another separate subscale was formed by co-workers/study colleagues (the latter subscale to be applied when necessary), all of them explaining a high percentage of variance. The rationale for separating the work subscale is merely operational: around half of the study population is either retired (elderly), or unemployed, or does not enter into the labor market. All the considered scales and subscales fitted better with the congeneric model of reliability, suggesting that, while consistent, they seem to be measured with different degrees of precision and different amounts of error. This would mean that the SOCQ definition, in terms of items and components, seems extensive enough in order to be referred to several interrelated facets (such us group membership, talking cliques, caring and supporting, trusting, laughing, shared values, close relationships), which might be studied in isolation in future works. This seems to be in agreement with the plurality of factors involved in other approaches, such as loneliness [79], emotional and social loneliness [68], perceived social support [67], and “social capital” [48, 54], providing a complementary psychosocial background to the latter notion.

The convergence of SOCQ with other constructs included in the study such as loneliness, psychological distress, and personality was satisfactorily high, demonstrating its validity and relevance by means of a number of significant correlations. Inverse relationships statistically significant were found between loneliness and the overall SOCQ, as well as with all the sociotype subscales. The same happened in the relationships between psychological distress and the SOCQ scale and subscales. It would reinforce the idea of a strong social network associated with less psychological distress (anxiety, depression) and reduced feelings of loneliness, as well as the idea of social/personal support perceived as a moderator between the sociotype and health variables [6, 7, 45, 57]. Interestingly, in the correlation between the SOCQ and personality dimensions, extraversion acquired a positive valence, while neuroticism and psychoticism were negatively correlated. The lie dimension correlated negatively with friends, and positively with acquaintances, which is reasonable in psychological terms and also seems to agree with “the logic of deceit and self-deception in human life” [102]. In general, the higher the sociotype scores, the better prospects regarding loneliness and psychological distress. Indeed the correlation values found were surprisingly elevated. But the interpretation of these correlations is far from direct, as always happens when causation is tentatively inferred from correlation. So, in order to facilitate further exploration from other points of view we have included the whole data gathered.

When using multivariate models that included personality traits, we observed that the different SOCQ components were differently connected to loneliness and psychological distress, but significant relationships were maintained as a whole and a large amount of variance was explained for both constructs. The regression model of loneliness was showing an important impairment of social networking in terms of family, friends, and acquaintances, as well as significant associations with personality traits such us extraversion, neuroticism, psychoticism and lie (negatively correlated in extraversion and lie, and positively in neuroticism and psychoticism). It may be interesting that lie’s higher scores correlate with lower scores in loneliness: the adaptive value of lies in social intercourse is well established, although complex tradeoffs between individual reputations and group subcultures are inevitably involved [43, 102]. Similarly, although less strongly, psychological distress was explained by impaired social networking in terms of family, friends, acquaintances, and also co-workers or study colleagues, involving the personal traits of extraversion and neuroticism—the first one negatively correlated, and the second positively.

It is also worth noting that a positive sociotype at work/study does not seem to influence on negative feelings of loneliness, for the deterioration of the other sociotype dimensions (family, friends, and acquaintances) acquires greater relevance in this case [47, 49, 103]. Conversely, deterioration of sociotype at work/study seems to greatly contribute to psychological distress, even gaining more relevance than the other sociotype dimensions [13, 60, 104]. A working hypothesis might be that strengthening overall sociotype dimensions (family, friends, acquaintances) would play a protective role against feelings of isolation, perhaps by perceived social support [103, 105]; while specifically strengthening the sociotype dimension at work/studies could be useful to prevent the psychological distress associated with chronic job stress. Therefore, improving the sociotype in the workplace through group dynamics [4] could be an effective strategy contributing to prevent, for instance, burnout syndrome [104]. As stated, other interpretations would be feasible, and the data of the study are freely available to tentatively support them (S1 Table).

Regarding the relationship between perceived social support [67] and sociotype, in spite of their superficial similarity, there is an important difference between them. The former has an implicit sense of dependence, of vulnerability, of counting with alien support for covering personal needs; while the sociotype refers to unmediated relationships, to spontaneous talking, to a sense of empowerment while the subject carries her/his relationships autonomously. Presumably, the degree of relationship between both constructs will strongly depend on the level of autonomy of the subject, e.g., in the age ranks of the elderly, both constructs will show more differences for relatively "young" elderly, while for the oldest segments (or “fourth age”) there will be more similarity. In any case, that interrelationship would imply a dedicated fieldwork (premature in the present developmental stage of the sociotype indicator).

In gender analysis, males showed a stronger social network in terms of coworkers or study colleagues, while in females the most important networking was in terms of family. This result is far from unexpected, given the deep cultural and social factors involved as well as the distinct relational strategies and reproductive interests [13, 44, 45]. In terms of age, we found significant differences among youth, midlife, maturity, and elderly life stages concerning the overall sociotype, as well as the subscales of friends, acquaintances, and work/study colleagues. It is interesting that the acquaintances subscale reaches the highest scores in the last stage (elderly) and the lowest in the first stage (youth); while for the general sociotype and the other subscales the highest scores appear in the first stage and the lowest scores appear in the last stage. It can be argued that for the elderly, friends and family gradually disappear from the relational scene, and their social interrelationship becomes progressively restricted to the casual and weak [51, 52, 106, 107]. However, alternative explanations would be available, for example it could be that the scale is optimally designed to capture the kinds of friends/family interactions that younger adults have more frequently. All we can say from the data is that mean ratings on the SOCQ are lower among older compared to younger adults for friends/family and higher among older compared to younger adults for acquaintances. Notwithstanding that, the family subscale did not show significant changes along the different stages of life, possibly due to the generational replacement occurring within the family network set. Future studies on the respective structures and conversation times involved will delimit the extent and interrelationships of these age-related phenomena. The perceived social self-satisfaction showed no differences according to sex or ages either.

As for limitations of the present empirical study, the main one was that it was reduced to just one country and to subjects sharing a relatively homogeneous culture—but it is hardly inevitable in a first exploration, and further multicultural studies are envisionned. Also, the intentionality of the sample, which nevertheless yielded strata balanced between all age groups (a variable of considerable importance in initially shaping the sociotype construct), and finally resulting in a considerable sample size above one thousand participants. Obviously, there is also the need of further separate studies by using adequate designs to test the essential sociotype determinants—mainly age, gender, personality, social environment, culture. As a first step in that direction, a quantitative study of the relational structures and estimated conversation times will be undertaken by the authors based on the other quantitative data gathered in this fieldwork (work in progress). Nevertheless, in the statistical analysis of the present study, all the procedures respected the true nature of the variables and complied with other metric assumptions necessary to carry out the various analyses.

The central message of this study should be understood within the *genotype-phenotype-sociotype* cord discussed at the Introduction [6, 7, 10–13]. The potential for social connection is in our genes, and it is in the development of this social potential where the integral mental and physical health of our bodies is ensconced. The problem to properly situate such a global construct within the very center of our social nature is that too many other factors and influences are crisscrossing thereby. Those confunding factors represent conceptual difficulties to disentangle, but at the same time they constitute the most important directions for future sociotype advancement.

Among those future directions, there is firstly the nature of the interpersonal bond and the different classes of social bonds [108, 109], which includes the bonding cognitive dynamics, the specific memory investment, and the asymmetric equivalences among bonds [4]; secondly, the centrality of conversation in the making and breaking of human bonds [25, 103]; thirdly, the inevitable gender differences in both social bonding and relational/reproductive interests [44]; and fourthly, the phenotype-sociotype tight interrelationship during the life course of each individual as well as the potential epigenetic consequences of individual failures in social environments [7]. Other directions closer to our times would be: the role of new communication technologies in revolutionizing the sociotype mix of individuals [59, 60, 110]; the traditional social and cultural schemes for work-leisure distribution and their present disruption [48, 54]; the importance of social networks in health and disease, and their potential role in the sustainability of the health care system [14]; the contemporary epidemics of loneliness and depression, particularly among the elderly, and the difficulties of the resocialization interventions [107]; and so on and so forth.

All those intractable problems and complicate circumstances of social life that surround the sociotype participate in its fluid conformation. In the extent to which the proposed pshychometric indicator could be properly delimited and diversified throughout future studies (to insist: the present study is but a first pilot step), an increasing number of applied topics might benefit from this new way of thinking.

## Conclusions

Operationally, the new indicator resulting from the present fieldwork seems to be valid and reliable, as well as closely associated with well validated metrics of loneliness and psychological distress. Reflecting the whole sociotype construct, the new psychometric indicator noticeably varies throughout the life course and circumstances of individuals, based on their gender and age, and adjusting to the different personal conditions of social networking. We think that the information already provided by this first operational definition around the sociotype construct, in spite of its preliminary nature, could be useful to guide the development and evaluation of programs aimed at improving and strengthening deteriorating social networks in people at risk, given their demographic characteristics (no family, no job, domestic violence, orphans, migrants, etc.) or depending on age (the vulnerability of children or young people, and especially the elderly).

## Supporting information

S1 Table Data of the study. Data obtained from the self-completed questionnaires of the survey.
(XLSX)

S1 File Sociotype questionnaire (SOCQ). English version of the "Sociotype Questionnaire" (SOCQ).
(DOC)

S2 File Interview guide (English). English version of the interview guide for the qualitative study.
(DOC)

S3 File Interview guide (Spanish). Spanish original of the interview guide for the qualitative study.
(DOC)

S4 File Guide of survey’s questions. English version of all the questions included in the survey.
(DOCX)

S5 File General Health Questionnaire (GHQ-12). Measurement of psicological distress.
(PDF)

S6 File Revised UCLA Loneliness Scale (RULS). Measurement of loneliness and social isolation.
(PDF)

S7 File Eysenck Personality Questionnaire-Revised (EPQ-R). Measurement of the major personality dimensions.
(PDF)
