# Supplementary material for: The “sociotype” construct: Gauging the structure and dynamics of human sociality
Source: PLoS One. 2017 Dec 14;12(12):e0189568. doi: 10.1371/journal.pone.0189568 (PMC5730176; doi:10.1371/journal.pone.0189568)
Supplement: S1 File — English version of the "Sociotype Questionnaire" (SOCQ). (DOC) [file pone.0189568.s002.doc]

**English version of the "Sociotype Questionnaire" (SOCQ)**

The following is a series of statements to asses the relationships with your family, friends, acquaintances and work/study colleagues. Read each statement carefully and mark with an X the option that best represents how you feel, what you do, and what you think about your social relationships. There are no right or wrong answers. In any case, please DO NOT LEAVE ANY STATEMENT UNANSWERED.

|  | **Never** | **Hardly**  **Ever** | **Sometimes** | **Often** | **Usually** | **Always** |
| --- | --- | --- | --- | --- | --- | --- |
| 1. I speak and relate with my family | O | O | O | O | O | O |
| 2. My family is important for me | O | O | O | O | O | O |
| 3. The family members care about me | O | O | O | O | O | O |
| 4. I have fun and laugh with my family | O | O | O | O | O | O |
| 5. I speak and relate with my friends | O | O | O | O | O | O |
| 6. I have friends to tell and share problems | O | O | O | O | O | O |
| 7. I consider important to maintain relationships with friends | O | O | O | O | O | O |
| 8. I have fun and laugh with my friends | O | O | O | O | O | O |
| 9. I speak and relate comfortably with acquaintances | O | O | O | O | O | O |
| 10. It costs me make conversation with people I do not know | O | O | O | O | O | O |
| 11. It is easy for me to win support from acquaintances | O | O | O | O | O | O |
| 12. Relations with my acquaintances are forced | O | O | O | O | O | O |
| 13. I speak and relate satisfactorily with my peers | O | O | O | O | O | O |
| 14. I have personal trust in my peers | O | O | O | O | O | O |
| 15. When talking with peers they take me into account | O | O | O | O | O | O |
| 16. I feel valued by my peers | O | O | O | O | O | O |

**Scoring**

Answers are scored from 0 (never) to 5 (always) for all items except nº 10 and 12 that have a reverse score from 5 (never) to 0 (always). General SOCQ covers all items, and is made up of 4 subscales: ‘family’ (items nº 1 to 4), ‘friends’ (items nº 5 to 8), ‘acquaintances’ (items nº 9 to 12), and ‘work/study colleagues’ (items nº 13 to 16).
