# Supplementary material for: The “sociotype” construct: Gauging the structure and dynamics of human sociality
Source: PLoS One. 2017 Dec 14;12(12):e0189568. doi: 10.1371/journal.pone.0189568 (PMC5730176; doi:10.1371/journal.pone.0189568)
Supplement: S2 File — English version of the interview guide for the qualitative study. (DOC) [file pone.0189568.s003.doc]

**SOCIOTYPE INTERVIEW GUIDE**

PARTICIPANT CODE: ­­­­­­­­­­­­­­­____________________

*(Directions for interviewer)*

Hello, good afternoon.

First of all I wanted to thank you for the interest you have shown in taking part in this study and for devoting part of your time to talking about these topics. My name is ____ and, as you know, we are here because we are conducting research on issues related to relationships between people.

We believe that your opinion is very important and, for that reason, I would like to encourage you to speak freely and to comment everything that you think opportune. All opinions are important and will be taken into account. It is about, spontaneously, talking on whatever things that are coming up.

As everything we say can be important and it is not possible to take note of everything, if you have no problem, we will record the conversation so that we can then listen to it more calmly.

In any case, I guarantee that everything will be treated anonymously and confidentially, so that you do not have to say your name or any other information that can identify you. These conversations usually last about an hour, but it will also depend a little on the things that we are talking about.

Do you have any questions or concerns?

1. I would like to start talking, for example, about what you think about the use of New Technologies (NT) as a way to maintain social relations.

*To explore:*

| Face-to-face interaction vs. NT |  |
| --- | --- |
| Expressive possibilities |  |
| Reflexivity vs. spontaneity |  |
| Virtual relationships |  |

2. How do you maintain your social relationships?

*To explore:*

| Communication channels |  |
| --- | --- |
| Positive aspects (advantages) |  |
| Associated difficulties (disadvantages) |  |
| Engagement in other social activities through speech |  |
| Satisfaction levels |  |

3. Which people do you usually interact with?

*To explore:*

| Types of relationships |  |
| --- | --- |
| Number of people |  |
| Quality of interactions |  |

4. How are your personal relationships?

*To explore:*

| What do you expect of them? |  |
| --- | --- |
| Satisfaction levels |  |
| Feelings related to the relationships |  |
| Feelings related to the channels |  |
