# Supplementary material for: The “sociotype” construct: Gauging the structure and dynamics of human sociality
Source: PLoS One. 2017 Dec 14;12(12):e0189568. doi: 10.1371/journal.pone.0189568 (PMC5730176; doi:10.1371/journal.pone.0189568)
Supplement: S3 File — Spanish original of the interview guide for the qualitative study. (DOC) [file pone.0189568.s004.doc]

**ENTREVISTA SOCIOTIPO**

PARTICIPANTE:

Hola, buenas tardes.

Antes de nada quería darte las gracias por el interés que has mostrado a la hora de participar en este estudio y por dedicar parte de tu tiempo a charlar sobre algunos temas. Mi nombre es Raquel y, como sabes, estamos aquí porque estamos llevando a cabo una investigación sobre temas relativos a las relaciones entre las personas.

Creemos que tu opinión es muy importante y, por eso, me gustaría animarte a hablar libremente y a comentar todo lo que te parezca oportuno. Todas las opiniones son importantes y van a ser tenidas en cuenta. Se trata de que vayamos hablando de las cosas que vayan surgiendo.

Como todo lo que comentemos va a ser importante y no es posible tomar nota de todo, si no tienes inconveniente, vamos a grabar la conversación para que luego podamos escucharla con más tranquilidad.

En cualquier caso, te garantizo que todo va a ser tratado de forma anónima y confidencial, de tal modo que no es necesario que digas tu nombre ni ningún otro dato que pueda identificarte. Estas conversaciones suelen durar aproximadamente una hora, pero también dependerá un poco de las cosas que vayamos hablando.

¿Tienes alguna pregunta o duda al respecto?

1. Me gustaría empezar a hablar, por ejemplo, sobre lo que piensas respecto a la utilización de las Nuevas Tecnologías como forma de mantener relaciones sociales.

Explorar:

| Interacción cara a cara vs. NT |  |
| --- | --- |
| Posibilidades expresivas |  |
| Reflexividad vs. espontaneidad |  |
| Relaciones virtuales |  |

2. ¿Cómo mantienes tus relaciones sociales?

Explorar:

| Canales de comunicación |  |
| --- | --- |
| Aspectos positivos |  |
| Dificultades asociadas |  |
| Dedicación a través del habla o de otras actividades |  |
| Niveles de satisfacción |  |

3. ¿Con qué personas sueles relacionarte de forma habitual?

Explorar:

| Tipos de relación |  |
| --- | --- |
| Cantidad de personas |  |
| Calidad de las interacciones |  |

4. ¿Cómo son tus relaciones personales?

Explorar:

| Qué esperas de ellas |  |
| --- | --- |
| Niveles de satisfacción |  |
| Sentimientos ligados a las relaciones |  |
| Sentimientos ligados a los canales |  |
