# Supplementary material for: The “sociotype” construct: Gauging the structure and dynamics of human sociality
Source: PLoS One. 2017 Dec 14;12(12):e0189568. doi: 10.1371/journal.pone.0189568 (PMC5730176; doi:10.1371/journal.pone.0189568)
Supplement: S4 File — English version of all the questions included in the survey. (DOCX) [file pone.0189568.s005.docx]

**SOCIOTYPE SURVEY**

**Guide of Questions**

SOCIODEMOGRAPHIC DATA (in yellow in the excel file)

- Sex

*[1= male, 2= female]*

- Age
- Civil Status

*[1= partner/married, 2= single, 3= separated/divorced, 4= widow/widower]*

- Connivance

*[1= alone, 2= partner, 3= partner and children, 4= other family, 5= friends, 6= residence]*

- Education

*[1= no studies, 2= primary, 3= high school, 4= university]*

- Employment

*[1= student, 2= unemployed, 3= employed, 4= retired]*

- Salary

*[1= <Minimum Wage (MW), 2= 1-2 MW, 3= 2-4 MW, 4= >4MW]*

1. SOCIOTYPE (in blue in the excel file)

Family dimension

1. I speak and relate with my family
2. My family is important for me
3. I find it difficult to get along with my family
4. The family members care about me
5. I have a sentimental partner who gives me support
6. I have fun and laugh with my family
7. I know how to deal with problems and conflicts in my family
8. I am satisfied with the status and way of life of my family

Friend dimension

1. I speak and relate with my friends
2. *I have friends to tell and share problems
3. I consider important to maintain relationships with friends
4. *With the rush and the stress I do not find time for my relations
5. I am open to new friendships
6. My friends consider I am a talkative person
7. I have fun and laugh with my friends
8. My friends have been useful to place myself socially

Work/Study Dimension

1. I speak and relate satisfactorily with my peers
2. I have personal trust in my peers
3. When talking with peers they take me into account
4. You also relate to them outside the educational / labor sphere
5. My activities at work (or study) are stimulating
6. I feel uncomfortable with teamwork, I prefer working individually
7. I feel valued by my peers
8. I am satisfied with the position reached, it corresponds to my authentic value

Acquaintances dimension

1. I speak and relate comfortably with acquaintances
2. *It costs me make conversation with people I do not know
3. It is easy for me to win support from acquaintances
4. *Relations with my acquaintances are forced
5. I prefer to speak to listen when relating to others
6. I like to introduce humor into my relationships with acquaintances
7. I am concerned about giving a good image to others
8. I prefer to relate to people of my social level or higher

Additional question

- Rate your personal satisfaction with social relationships from 0 to 100

*Correction values:*

*[1= Never, 2= Hardly ever, 3= Sometimes, 4= Often, 5= Usually, 6= Always]*

*The questions marked as (*) have an inverted punctuation.*

2. GHQ-12 (in orange in the excel file)

1. Have you recently been able to concentrate on what you’re doing?
2. Have you recently lost much sleep over worry?
3. Have you recently felt you were playing a useful part in things?
4. Have you recently felt capable of making decisions about things?
5. Have you recently felt constantly under strain?
6. Have you recently felt you couldn’t overcome your difficulties?
7. Have you recently been able to enjoy your normal day-to-day activities?
8. Have you recently been able to face up to your problems?
9. Have you recently been feeling unhappy and depressed?
10. Have you recently been losing confidence in yourself?
11. Have you recently been thinking of yourself as a worthless person?
12. Have you recently been feeling reasonably happy, all things considered

3. UCLA (in violet in the excel file)

1. I feel in tune with the people around me
2. I lack companionship
3. There is no-one I can turn to
4. I do not feel alone
5. I feel part of a group of friends
6. I have a lot in common with the people around me
7. I am no longer close to anyone
8. My interests and ideas are not shared by those around me
9. I am an outgoing person
10. There are people I feel close to
11. I feel left out
12. My social relationships are superficial
13. No-one really knows me well
14. I feel isolated from others
15. I can find companionship when I want it
16. There are people who really understand me
17. I am unhappy being so withdrawn
18. People are around me but not with me
19. There are people I can talk to
20. There are people I can turn to

4. EYSENCK (in green in the excel file)

1. Do you have many different hobbies?
2. Do you stop to think things over before doing anything?
3. Does your mood often go up and down?
4. Are you a talkative person?
5. Do you ever feel ‘just miserable’ for no reason?
6. Do you give money to charities?
7. Were you ever greedy by helping yourself to more than your share of anything?
8. Are you rather lively?
9. Would it upset you a lot to see a child or an animal suffer?
10. Do you often worry about things you should not have said or done?
11. If you say you will do something, do you always keep your promise no matter how inconvenient it might be?
12. Can you usually let yourself go and enjoy yourself at a lively party?
13. Are you an irritable person?
14. Do you take much notice of what people think?
15. Have you ever blamed someone for doing something you knew was really your fault?
16. Are your feelings easily hurt?
17. Are all your habits good and desirable ones?
18. Do you tend to keep in the background on social occasions?
19. Do you often feel ‘fed-up’?
20. Have you ever taken anything (even a pin or button) that belonged to someone else?
21. For you, the boundaries between what is right and what is wrong are less clear than for most people?
22. Do you like going out a lot?
23. Do you prefer to go your own way rather than act by the rules?
24. Do you enjoy hurting people you love?
25. Are you often troubled about feelings of guilt?
26. Do you sometimes talk about things you know nothing about?
27. Do you prefer reading to meeting people?
28. Would you call yourself a nervous person?
29. Do you have many friends?
30. Have you faced your parents frequently?
31. Are you a worrier?
32. As a child did you do as you were told immediately and without grumbling?
33. Have you often gone against your parents’ wishes?
34. Do you worry about awful things that might happen?
35. Have you ever broken or lost something belonging to someone else?
36. Do you usually take the initiative in making new friends?
37. Personal desires are above social norms
38. Would you call yourself tense or ‘highly-strung’?
39. Are you mostly quiet when you are with other people?
40. Do you think marriage is old-fashioned and should be done away with?
41. Are you more easy-going about right and wrong than most people?
42. Can you easily get some life into a rather dull party?
43. Do you worry about your health?
44. Have you ever said anything bad or nasty about anyone?
45. Do you enjoy co-operating with others?
46. Do you like telling jokes and funny stories to your friends?
47. Do most things taste the same to you?
48. As a child were you ever cheeky to your parents?
49. Do you like mixing with people?
50. Does it worry you if you know there are mistakes in your work?
51. Do you always wash before a meal?
52. Do you nearly always have a ‘ready answer’ when people talk to you?
53. Have you often felt listless and tired for no reason?
54. Have you ever cheated at a game?
55. Do you like doing things in which you have to act quickly?
56. Is (or was) your mother a good woman?
57. Do you often make decisions on the spur of the moment?
58. Do you often feel life is very dull?
59. Have you ever taken advantage of someone?
60. Do you worry a lot about your looks?
61. Do you think people spend too much time safeguarding their future with savings and insurance?
62. Have you ever wished that you were dead?
63. Would you dodge paying taxes if you were sure you could never be found out?
64. Can you get a party going?
65. Do you try not to be rude to people?
66. Do you worry too long after an embarrassing experience?
67. Do you generally ‘look before you leap’?
68. Do you suffer from ‘nerves’?
69. Do you often feel lonely?
70. Do you always practice what you preach?
71. Are you easily hurt when people find fault with you on the work you do?
72. Is it better to follow society’s rules than go your own way?
73. Have you ever been late for an appointment or work?
74. Do you like plenty of bustle and excitement around you?
75. Do you frequently improvise decisions based on the situation?
76. Are you sometimes bubbling over with energy and sometimes very sluggish?
77. Do you sometimes put off until tomorrow what you ought to do today?
78. Do other people think of you as being very lively?
79. Do people tell you a lot o flies?
80. Are you touchy about some things?
81. Are you always willing to admit it when you have made a mistake?
82. When your temper rises, do you find it difficult to control?
83. Do you think insurance plans are a good idea?

*[1= Yes, 2= No]*
