# Supplementary material for: The “sociotype” construct: Gauging the structure and dynamics of human sociality
Source: PLoS One. 2017 Dec 14;12(12):e0189568. doi: 10.1371/journal.pone.0189568 (PMC5730176; doi:10.1371/journal.pone.0189568)
Supplement: S5 File — Measurement of psicological distress. (PDF) [file pone.0189568.s006.pdf]

Short General Health Questionnaire (GHQ 12)

Have you recently?

1.	Been able to concentrate on what you're doing?	Better than usual	Same as usual	Less than usual	Much less than usual
2.	Lost much sleep over worry?	Not at all	No more than usual	Rather more than usual	Much more than usual
3.	Felt you were playing a useful part in things?	More so than usual	Same as usual	Less useful than usual	Much less useful
4.	Felt capable of making decisions about things?	More so than usual	Same as usual	Less so than usual	Much less capable
5.	Felt constantly under strain?	Not at all	No more than usual	Rather more than usual	Much more than usual
6.	Felt you couldn't overcome your difficulties?	Not at all	No more than usual	Rather more than usual	Much more than usual
7.	Been able to enjoy your normal day-to-day activities?	More so than usual	Same as usual	Less so than usual	Much less than usual
8.	Been able to face up to your problems?	More so than usual	Same as usual	Less so than usual	Much less able
9.	Been feeling unhappy and depressed?	Not at all	No more than usual	Rather more than usual	Much more than usual
10.	Been losing confidence in yourself?	Not at all	No more than usual	Rather more than usual	Much more than usual
11.	Been thinking of yourself as a worthless person?	Not at all	No more than usual	Rather more than usual	Much more than usual
12.	Been feeling reasonably happy, all things considered	More so than usual	About same as usual	Less so than usual	Much less than usual;
