# Supplementary material for: The “sociotype” construct: Gauging the structure and dynamics of human sociality
Source: PLoS One. 2017 Dec 14;12(12):e0189568. doi: 10.1371/journal.pone.0189568 (PMC5730176; doi:10.1371/journal.pone.0189568)
Supplement: S6 File — Measurement of loneliness and social isolation. (PDF) [file pone.0189568.s007.pdf]

Revised UCLA Loneliness Scale

Indicate how often you feel the way described in each statement using the following scale:

4 = "I feel this way ***often***."
3 = "I feel this way ***sometimes***."
2 = "I feel this way ***rarely***."
1 = "I have ***never*** felt this way."

1. I feel in tune with the people around me	
2. I lack companionship	
3. There is no-one I can turn to.	
4. I do not feel alone.	
5. I feel part of a group of friends.	
6. I have a lot in common with the people around me.	
7. I am no longer close to anyone.	
8. My interests and ideas are not shared by those around me.	
9. I am an outgoing person.	
10. There are people I feel close to.	
11. I feel left out.	
12. My social relationships are superficial.	
13. No-one really knows me well.	
14. I feel isolated from others.	
15. I can find companionship when I want it.	
16. There are people who really understand me.	
17. I am unhappy being so withdrawn.	
18. People are around me but not with me.	
19. There are people I can talk to.	
20. There are people I can turn to.	
