# Supplementary material for: The “sociotype” construct: Gauging the structure and dynamics of human sociality
Source: PLoS One. 2017 Dec 14;12(12):e0189568. doi: 10.1371/journal.pone.0189568 (PMC5730176; doi:10.1371/journal.pone.0189568)
Supplement: S7 File — Measurement of the major personality dimensions. (PDF) [file pone.0189568.s008.pdf]

Person. individ. Diff. Vol. 6, No. 1, pp. 21–29, 1985
Printed in Great Britain. All rights reserved

0191-8869/85 $3.00+0.00
Copyright © 1985 Pergamon Press

A REVISED VERSION OF THE PSYCHOTICISM SCALE

S. B. G. EYSENCK, H. J. EYSENCK and PAUL BARRETT
Department of Psychology, Institute of Psychiatry, De Crespigny Park, Denmark Hill, London SE5 8AF, England

(*Received* 28 *June* 1984)

Summary—In view of certain psychometric deficiencies of the original Psychoticism scale, an attempt was made to improve the scale by adding new items. It was attempted to increase the internal reliability of the scale, improve the shape of the distribution and increase the mean and variance score. Two different studies are discussed. Reliabilities are now somewhat improved, distributions are closer to normal and mean scores are higher than on the old scale. Four new short 12-item scales for the measurement of P, E, N and L are also given.

INTRODUCTION

The publication of *Psychoticism as a Dimension of Personality* (Eysenck and Eysenck, 1976) and the Eysenck Personality Questionnaire (EPQ; Eysenck and Eysenck, 1975) was accompanied by numerous criticisms of the psychometric properties of the P scale (Block, 1977a, b; Bishop, 1977), as well as other aspects of the concept itself; some of these were answered by Claridge and Birchall (1978), Eysenck (1977) and Eysenck and Eysenck (1977). A summary of the available evidence to date on all the points has been presented by Claridge (1981), and there is no intention here to discuss these issues any further. Our intention is to try and improve what are undoubtedly psychometric weaknesses in the P scale of the EPQ, by designing new items, based on the development of the original concept, testing the relevance of these items by new factor-analytic studies and construct improved questionnaires in the hope of improving upon the original version of the P scale.

There are three major faults in the original P scale which, while not apparently interfering too much with its validity in group comparisons, made individual application rather hazardous. The first of these faults is the *low reliability* of the scale, 0.74 for males and 0.68 for females. (These are internal reliabilities; test–retest reliabilities are rather higher, viz. 0.83 and 0.71.) The second fault is the *low range of scoring*, with means of 3.78 for males and 2.63 for females. The fact that standard deviations were almost identical with means (3.09 and 2.36) indicates the third fault, namely the *grossly skewed distribution* of scores, which almost resembled a Poissonian distribution. The fact that in spite of these faults the scale was found to behave very consistently and predictably (Claridge, 1981) suggests that the validity was not depressed too much by these psychometric faults, but clearly improvements should be made if possible.

SAMPLES AND METHODS

Two studies were, in fact, carried out to this end. The first (A) used a 90-item questionnaire which contained the EPQ P scale plus possible new P items, together with 12 E and 13 N items selected from the EPQ. There were no L-scale items. The sample tested consisted of 384 males and 290 females, whose ages ranged from 17 to 70 yr, their respective age means being 40.01 ± 14.39 and 37.99 ± 14.47 yr. Sample A was collected by random approach in the street and by house-to-house circulation of questionnaires.

The second sample (B) used a 117-item questionnaire which contained all the 90 items of the EPQ plus almost all the possible new P items used for the questionnaire of Study A. This sample was collected in a more orthodox manner, with groups of students, teachers and other willing and varied *S*s being approached to complete the questionnaire and returned by post. There were 408 males and 494 females aged 38.44 ± 17.67 and 31.80 ± 15.84 yr, respectively, the range being the same as in Study A.

In both studies the items were intercorrelated separately for males and females and the resulting matrices factor-analysed by principal components. In Study A, three factors were extracted and rotated through Varimax and then Promax, these being identified as P, E and N. In Study B, however, four factors were extracted and were similarly rotated using Direct Oblimin rotation. These were clearly identified as P, E, N and the L scale.

Both sets of loadings were scrutinized for suitable P items to improve the original scale. Table 1 gives the 32 items which were finally chosen to make up the P scale of the EPQ—Revised (EPQ-R). It will be seen that only the P loadings are given for Sample A, but all P, E, N and L loadings are given for Sample B. This is because of the few E and N items in Study A, and the complete absence of L items.

Table 1. Factor loadings on P for Sample A and on P, E, N and L for Sample B for items on EPQ-R

No. on 100-item EPQ-R	MALES Sample A (n = 290)	MALES Sample B (n = 408)				FEMALES Sample A (n = 384)	FEMALES Sample B (n = 494)			
	P	P	E	N	L	P	P	E	N	L
−2	−0.34	−0.38	−0.11	−0.02	0.14	−0.19	−0.25	−0.14	−0.01	0.22
−5	−0.36	−0.42	0.18	0.17	−0.03	−0.30	−0.32	0.12	0.26	−0.09
−7	−0.43	−0.32	−0.18	0.17	0.11	−0.36	−0.30	−0.11	0.24	0.18
−9	−0.41	−0.33	−0.01	−0.07	−0.02	−0.35	−0.27	−0.06	−0.04	−0.02
−12	−0.31	−0.47	0.05	0.15	−0.02	−0.15	−0.26	0.01	−0.01	−0.20
−14	−0.33	−0.18	−0.04	0.16	0.23	−0.06	−0.21	−0.09	0.17	0.08
−18	−0.38	−0.28	0.08	−0.05	0.25	−0.38	−0.43	0.08	0.05	0.31
−21	−0.48	−0.25	0.26	0.08	0.24	−0.17	−0.44	0.16	0.13	0.12
25	0.26	0.30	0.13	0.08	−0.22	0.32	0.41	0.07	0.07	−0.19
29	0.47	0.21	0.01	−0.01	−0.22	0.51	0.39	−0.02	−0.11	−0.11
30	0.46	0.30	0.03	0.08	−0.01	0.24	0.32	0.17	0.18	0.16
34	0.31	0.29	0.11	0.25	0.11	0.32	0.28	0.11	0.25	0.15
37	0.40	0.37	0.20	0.03	−0.10	0.31	0.31	0.18	0.08	−0.05
−41	−0.55	−0.37	0.26	0.05	0.21	−0.26	−0.44	0.16	0.17	0.18
42	0.26	0.25	0.12	0.18	−0.17	0.42	0.39	0.08	−0.01	−0.14
48	0.33	0.43	−0.04	0.01	0.12	0.47	0.45	−0.08	0.08	0.07
50	0.34	0.22	0.04	−0.04	−0.09	0.40	0.32	0.06	−0.06	−0.01
−54	−0.46	−0.37	0.36	−0.07	0.04	−0.31	−0.33	0.12	−0.02	−0.10
56	0.05	0.39	−0.08	−0.03	0.18	0.18	0.34	0.02	0.18	0.31
−59	−0.53	−0.42	−0.12	0.17	0.09	−0.34	−0.39	−0.09	0.10	−0.02
−64	−0.27	−0.19	−0.07	0.04	0.09	−0.22	−0.32	0.03	0.03	0.05
−68	−0.26	−0.27	0.06	−0.08	−0.20	−0.17	−0.32	0.02	−0.10	−0.27
73	0.32	0.30	0.10	0.30	0.12	0.29	0.40	0.08	0.20	0.26
75	0.44	0.32	0.08	0.02	−0.07	0.35	0.30	0.08	−0.04	−0.03
−79	−0.51	−0.39	−0.02	0.04	0.11	−0.31	−0.34	−0.06	−0.04	0.11
−81	−0.27	−0.27	−0.16	−0.05	0.27	−0.20	−0.31	−0.25	−0.07	0.24
−85	−0.19	−0.40	0.01	−0.29	−0.25	−0.44	−0.20	0.14	−0.25	−0.10
−88	−0.50	−0.19	0.11	−0.02	0.26	−0.46	−0.37	0.07	0.08	0.19
91	0.36	0.53	0.17	0.16	0.09	0.36	0.44	0.02	0.19	0.24
95	0.14	0.38	0.19	0.26	0.20	0.33	0.26	0.03	0.22	0.14
−96	−0.41	−0.34	−0.04	−0.05	−0.06	−0.36	−0.38	0.06	0.04	−0.01
−99	−0.29	−0.36	−0.02	0.04	0.04	−0.20	−0.14	−0.04	−0.03	−0.14

RESULTS

Although Sample A were somewhat older than Sample B (especially the females), and the collection of the data was somewhat different, the P-scale means and standard deviations were pooled. These are given, for different age groups, in Table 2 and contain *S*s from Samples A and B combined for P, but only from Sample B for E, N and L. However, just as a matter of interest, Table 3 gives the actual means and standard deviations on P obtained by each of the age groups in Samples A and B, respectively.

The scoring key on which these means were calculated is given in Table 4, the item numbers referring to questions on the 100 item EPQ-R given in Appendix 1. There are now 32 items on the new P scale, i.e. 7 more than in the EPQ P scale and very slight changes to E and N were made so that the former has 23 and the latter 24 items, leaving L with the original 21 items. The total number of items, therefore, is now 100.

Table 2. Means and standard deviations of P, E, N and L for different age groups on the EPQ-R

Age group (yr)	Samples A + B			Sample B						
		P			E		N		L	
	n	$\bar{X}$	SD	n	$\bar{X}$	SD	$\bar{X}$	SD	$\bar{X}$	SD
					Males					
16–20	120	9.57	5.26	108	15.97	5.26	11.12	5.68	5.37	4.18
21–30	148	8.65	4.56	64	14.50	5.64	11.08	5.37	5.53	3.39
31–40	117	6.69	3.58	53	11.92	5.67	11.92	5.70	6.66	3.59
41–50	107	7.00	4.65	55	11.91	5.09	11.22	5.95	7.04	3.87
51–60	110	5.28	3.59	69	8.94	5.75	9.43	6.27	9.14	4.29
61–70	91	4.87	3.55	59	8.68	5.71	8.32	5.07	10.05	3.65
Total	693	7.19	4.60	408	12.51	6.00	10.54	5.81	7.10	4.28
					Females					
16–20	203	7.06	4.11	161	15.47	4.99	14.03	4.85	5.45	3.25
21–30	256	6.20	3.86	159	14.17	4.68	12.53	4.78	6.33	3.82
31–40	135	5.87	3.72	38	13.55	4.93	11.71	4.94	6.79	3.74
41–50	109	4.62	3.05	50	12.36	4.95	10.94	5.92	8.02	3.88
51–60	102	4.05	3.21	45	13.62	5.47	11.31	5.36	8.82	3.97
61–70	73	4.19	3.26	41	12.15	5.08	9.98	5.51	11.20	3.09
Total	878	5.73	3.85	494	14.14	5.06	12.47	5.22	6.88	3.97

Table 3. Means and standard deviations for the P scale only for Samples A, B and (A + B) combined

Age group (yr)	Sample B			Sample A			Samples B + A		
	n	$\bar{X}$	SD	n	$\bar{X}$	SD	n	$\bar{X}$	SD
					Males				
16–20	108	9.31	5.06	12	11.92	6.36	120	9.57	5.26
21–30	64	9.08	4.63	84	8.32	4.48	148	8.65	4.56
31–40	53	7.19	3.44	64	6.27	3.64	117	6.69	3.58
41–50	55	7.98	4.13	52	5.96	4.94	107	7.00	4.65
51–60	69	5.71	3.85	41	4.56	2.96	110	5.28	3.59
61–70	59	5.34	3.60	32	4.00	3.28	91	4.87	3.55
Total	408	7.63	4.57	285	6.55	4.58	693	7.19	4.60
					Females				
16–20	161	6.98	4.15	42	7.36	3.94	203	7.06	4.11
21–30	159	6.30	4.16	97	6.04	3.30	256	6.20	3.86
31–40	38	6.61	3.90	97	5.58	3.61	135	5.87	3.72
41–50	50	5.12	3.27	59	4.19	2.77	109	4.62	3.05
51–60	45	4.80	3.16	57	3.46	3.13	102	4.05	3.21
61–70	41	5.00	3.48	32	3.16	2.61	73	4.19	3.26
Total	494	6.18	4.00	384	5.16	3.56	878	5.73	3.85

Table 4. Scoring key for the EPQ-R (items numbered as for the 100-item questionnaire)

(P)

YES: 25, 29, 30, 34, 37, 42, 48, 50, 56, 73, 75, 91, 95
NO: 2, 5, 7, 9, 12, 14, 18, 21, 41, 54, 59, 64, 68, 79, 81, 85, 88, 96, 99

(32)

(E)

YES: 1, 6, 11, 16, 20, 28, 36, 40, 45, 51, 55, 58, 61, 63, 67, 69, 72, 78, 90, 94
NO: 24, 33, 47

(23)

(N)

YES: 3, 8, 13, 17, 22, 26, 31, 35, 38, 43 46, 52, 60, 65, 70, 74, 76, 80, 83, 84, 87, 92, 97, 100

(24)

(L)

YES: 15, 23, 39, 62, 86, 98
NO 4, 10, 19, 27, 32, 44, 49, 53, 57, 66, 71, 77, 82, 89, 93

(21)

It should be mentioned that there were 6 items from the original 25-item EPQ P scale, which have been omitted from the present revised scale. These were:

"Do you lock up your house carefully at night?"
"Do you believe insurance schemes are a good idea?"
"Do people who drive carefully annoy you?"
"When you catch a train do you often arrive at the last minute?"
"Do your friendships break up easily without it being your fault?"

and

"Do you sometimes like teasing animals?"

The main reason for these omissions was the lack of consistent loadings on the P factor.

However, 13 new P items were incorporated into the P scale which, along with 19 of the original EPQ P scale items now totals 32 items. In addition, two new E items warranted inclusion in the new E scale and one extra N item was added to the N scale, leaving L at the original 21 items.

Reliabilities (α-coefficients) and intercorrelations of the scales are given in Table 5. It will be noted that separate reliabilities for P are given for Samples A and B. For interest, the correlations between the new P scale (EPQ-R) and that of the EPQ were calculated and the values were 0.88 for males and 0.81 for females (Sample B only).

Finally, it seemed desirable to try to devise a short scale of the EPQ-R for use when time is very limited. Twelve items were chosen from each of the scales and the short scale questionnaire is given in Appendix 2. The means and standard deviations of all the age groups are given in Table 6. Again, as with the longer scale, Table 6 gives the P means and standard deviations of Samples A and B pooled, while Table 7 shows the individual age group means for the separate samples on P.

Reliabilities for both samples are given in Table 8 and also intercorrelations of the scales for Sample B. The scoring key on Table 9 gives item numbers to correspond with the 48-item short scale given in Appendix 2.

Table 5. Reliabilities of P (Sample A), and P, E, N and L (Sample B) (intercorrelations of P, E, N and L for Sample B only)

	Reliabilities Sample A	Sample B				*Intercorrelations (Sample B only)*		
	P	P	E	N	L		Males	Females
Males	0.81	0.78	0.90	0.88	0.82	PE	0.23	0.14
Females	0.73	0.76	0.85	0.85	0.79	PN	0.19	0.06
						PL	−0.34	−0.16
						EN	0.02	−0.07
						EL	−0.32	−0.19
						NL	−0.25	−0.26

Table 6. Means and standard deviations of P, E, N and L for different age groups on the short scale of the EPQ-R

Age group (yr)	Samples (A + B)			Sample B							
		P			E		N		L		
	n	$\bar{X}$	SD	n	$\bar{X}$	SD	$\bar{X}$	SD	$\bar{X}$	SD
				Males						
16–20	120	4.16	2.43	108	8.16	3.30	5.03	3.41	2.69	2.61
21–30	148	3.57	2.29	64	7.42	3.44	5.17	3.35	2.92	2.00
31–40	117	2.82	1.84	53	6.40	3.85	5.75	3.46	3.58	2.28
41–50	107	2.93	2.18	55	6.18	3.34	5.24	3.41	3.98	2.51
51–60	110	2.28	1.61	69	4.48	3.69	4.49	3.70	5.16	2.83
61–70	91	2.36	2.06	59	4.24	3.40	4.14	3.04	5.61	2.36
Total	693	3.08	2.20	408	6.36	3.80	4.95	3.44	3.86	2.71
				Females						
16–20	203	2.79	1.95	161	8.40	3.23	6.66	3.05	2.75	2.03
21–30	256	2.56	1.95	159	7.60	3.02	5.93	2.89	3.34	2.41
31–40	135	2.37	1.80	38	7.37	3.18	5.50	2.92	3.76	2.38
41–50	109	2.05	1.63	50	6.48	3.20	5.06	3.37	4.26	2.46
51–60	102	1.72	1.67	45	7.22	3.64	5.36	3.44	4.96	2.70
61–70	73	1.76	1.74	41	6.46	3.24	4.78	3.28	6.56	2.14
Total	878	2.35	1.88	494	7.60	3.27	5.90	3.14	3.69	2.55

Table 7. Means and standard deviations for the short-scale EPQ-R P scale only for Samples A, B and (A + B) combined

Age group (yr)	Sample B			Sample A			Samples B + A		
	n	$\bar{X}$	SD	n	$\bar{X}$	SD	n	$\bar{X}$	SD
					Males				
16-20	108	4.05	2.27	12	5.17	3.39	120	4.16	2.43
21-30	64	3.83	2.26	84	3.38	2.29	148	3.57	2.29
31-40	53	3.23	1.82	64	2.48	1.79	117	2.82	1.84
41-50	55	3.44	2.03	52	2.40	2.21	107	2.93	2.18
51-60	69	2.38	1.72	41	2.12	1.38	110	2.28	1.61
61-70	59	2.61	2.16	32	1.91	1.76	91	2.36	2.06
Total	408	3.33	2.18	285	2.73	2.19	693	3.08	2.20
					Females				
16-20	161	2.84	1.99	42	2.60	1.75	203	2.79	1.95
21-30	159	2.74	2.08	97	2.27	1.67	256	2.56	1.95
31-40	38	2.68	2.08	97	2.25	1.66	135	2.37	1.80
41-50	50	2.34	1.66	59	1.80	1.57	109	2.05	1.63
51-60	45	1.98	1.54	57	1.51	1.74	102	1.72	1.67
61-70	41	2.20	1.88	32	1.19	1.33	73	1.76	1.74
Total	494	2.61	1.97	384	2.02	1.69	878	2.35	1.88

Table 8. Short-scale reliabilities of P (Sample A) and P, E, N and L (Sample B) (short-scale intercorrelations of P, E, N and L for Sample B only)

	Reliabilities Sample A	Sample B					*Intercorrelations (Sample B only)*	
	P	P	E	N	L		Males	Females
Males	0.68	0.62	0.88	0.84	0.77	PE	0.07	0.04
Females	0.51	0.61	0.84	0.80	0.73	PN	0.14	0.04
						PL	−0.23	−0.13
						EN	−0.09	−0.10
						EL	−0.21	−0.12
						NL	−0.16	−0.19

Table 9. Short-scale EPQ-R scoring key

Ⓟ		
YES:	10, 14, 22, 31, 39	
NO:	2, 6, 18, 26, 28, 35, 43	(12)
Ⓔ		
YES:	3, 7, 11, 15, 19, 23, 32, 36, 44, 48	(12)
NO:	27, 41	
Ⓝ		
YES:	1, 5, 9, 13, 17, 21, 25, 30, 34, 38, 42, 46	(12)
Ⓛ		
YES:	4, 16, 45	
NO:	8, 12, 20, 24, 29, 33, 37, 40, 47	(12)

DISCUSSION

Since, admittedly, the P scale of the EPQ suffered from several psychometric shortcomings, an attempt has now been made to rectify some of these. The three main areas of criticism have been listed in the Introduction as: (1) low reliability, (2) low range of scoring and (3) grossly skewed distribution.

The reliabilities (see Table 5) are 0.78 for males and 0.76 for females which contrasts with 0.74 for males and 0.68 for females as quoted in the EPQ manual (Eysenck and Eysenck, 1975). Although this is still not as high as the reliabilities achieved for E, N and L, it must be remembered that the P scale taps several different facets (hostility, cruelty, lack of empathy, non-conformism etc.) which

may hold reliabilities lower than would be true of a scale like E which comprises largely sociability and activity items only (Cattell and Tsujioka, 1964). At any rate, the new reliabilities are now acceptable and are certainly an improvement on those of the EPQ P scale.

The low range of scoring of the 25-item P scale was a great disadvantage in that means of 3.78 ± 3.09 for males and 2.63 ± 2.36 for females, quoted in the manual of the EPQ, gave very little room for differentiation. The nature of the scale, unfortunately, is such that most discriminating items are somewhat 'way out' for most people. The original PEN P scale contained even more paranoid and schizoid type items than the EPQ P scale, and some criticism of this latter scale from researchers like Claridge (1981) dwells on the better discrimination of the old PEN P scale as far as schizophrenics are concerned. Hopefully the present changes of the P scale will not further reduce the usefulness of the EPQ-R for work with schizophrenics, but this remains to be seen when the scale is applied to clinical groups in due course. Meanwhile, Table 2, shows means of 7.19 ± 4.60 for males and 5.73 ± 3.85 for females; this contrasts with 4.15 ± 3.42 for males and 2.97 ± 2.59 for females when the same groups are scored for P on the original 25 items of the EPQ. This difference seems a considerable improvement giving greater leeway in scoring.

Finally, the question of skewness and kurtosis of distribution was investigated. Since all 25 original EPQ P scale items are contained in the questionnaires used in both Studies A and B, it was possible to compute the moments for this scale as well as for the 32-item new P scale form. Both distributions, of combined Studies A and B *S*s, were plotted as histograms and are given in Figs 1 and 2. It can be seen that there is a somewhat improved skewness in the new distribution, especially so for the females, and this is reflected in the drop in both skewness and kurtosis values listed in the figures. It would be unreasonable to expect a dramatic normalizing of the distribution for the P scale, since the scale itself must inevitably, by its very nature, constitute some departure from normality. This is so, not only by reason of the type of items which differentiate high and low P scorers, but also highlights a proven hallmark of high P scorers to be uncooperative as far as helping researchers with questionnaire replies. "Did you mind filling in this form?" gives consistent loadings on P and suggests that high P scorers would be much less likely to complete a personality questionnaire than low P scorers. Thus, however improved the P scale, it seems likely to be doomed to a slightly J-shaped distribution as long as high P scorers are able to decline to cooperate.

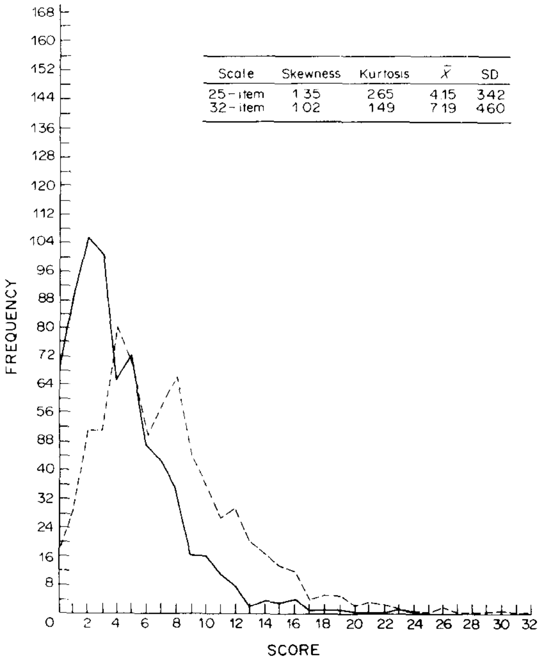

Fig. 1. Distributions of combined Studies A + B for males (n = 693). ——25-item scale; - - - 32-item scale.

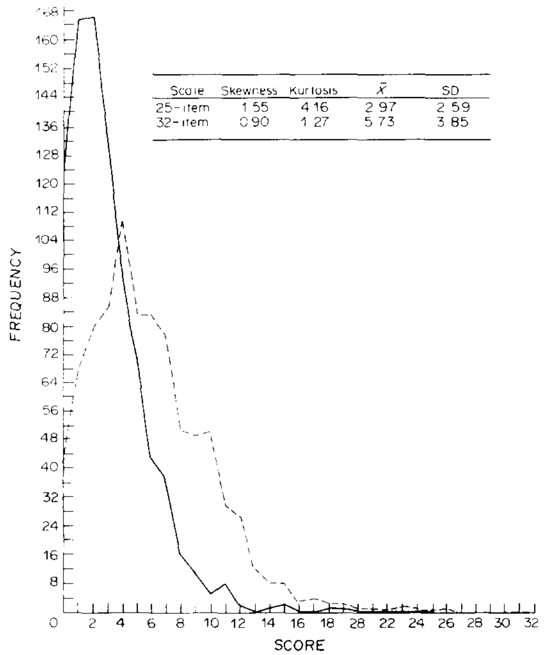

Fig. 2. Distributions of combined Studies A + B for females (n = 878). ——25-item scale; - - - 32-item scale.

Nevertheless, we feel that all these areas of improvement which we set out to tackle, have yielded some success and, hopefully, researchers may try the new questionnaire out in future studies.

Acknowledgements—We are indebted to Dr G. Easting, Mr P. R. Pearson and Dr C. Gosselin for help in the collection of the data. Also, our thanks to Mrs Maeve Denby and the British Humanist Association for contributing *Ss* to our data. Our thanks also to Jackie Marshall for computational help.

REFERENCES

Bishop D. V. M. (1977) The P scale and psychosis. *J. abnorm. Psychol.* **86,** 127–134.
Block J. (1977a) P scale and psychosis: continued concerns. *J. abnorm. Psychol.* **85,** 431–434.
Block J. (1977b) The Eysencks and psychoticism. *J. abnorm. Psychol.* **86,** 656–.
Cattell R. B. and Tsujioka B. (1964) The importance of factor trueness and of validity versus homogeneity and orthogonality in test scales. *Educ. psychol Measur.* **24,** 3–30.
Claridge G. (1981) Psychoticism. In *Dimensions of Personality* (Edited by Lynn R.), pp. 79–110. Pergamon Press, New York.
Claridge G. and Birchall P. (1978) Bishop, Eysenck, Block and psychoticism. *J. abnorm. Psychol.* **87,** 664–668.
Eysenck H. J. (1977) Psychosis and psychoticism: a reply to Bishop. *J. abnorm. Psychol.* **86,** 427–430.
Eysenck H. J. and Eysenck S. B. G. (1975) *Manual of the Eysenck Personality Questionnaire.* Hodder & Stoughton, London.
Eysenck H. J. and Eysenck S. B. G. (1976) *Psychoticism as a Dimension of Personality.* Hodder & Stoughton, London.
Eysenck H. J. and Eysenck S. B. G. (1977) Block and psychoticism. *J. abnorm. Psychol.* **86,** 651–652.

APPENDIX 1

EPQ-R

Occupation .... Age .... Sex ....

Instructions: Please answer each question by putting a circle around the 'YES' or the 'NO' following the question. There are no right or wrong answers, and no trick questions. Work quickly and do not think too long about the exact meaning of the questions.

PLEASE REMEMBER TO ANSWER EACH QUESTION

1. Do you have many different hobbies? .... YES NO
2. Do you stop to think things over before doing anything? .... YES NO
3. Does your mood often go up and down? .... YES NO
4. Have you ever taken the praise for something you knew someone else had really done? .... YES NO
5. Do you take much notice of what people think? .... YES NO
6. Are you a talkative person? .... YES NO
7. Would being in debt worry you? .... YES NO
8. Do you ever feel 'just miserable' for no reason? .... YES NO
9. Do you give money to charities? .... YES NO
10. Were you ever greedy by helping yourself to more than your share of anything? .... YES NO
11. Are you rather lively? .... YES NO
12. Would it upset you a lot to see a child or an animal suffer? .... YES NO
13. Do you often worry about things you should not have done or said? .... YES NO
14. Do you dislike people who don't know how to behave themselves? .... YES NO
15. If you say you will do something, do you always keep your promise no matter how inconvenient it might be? .... YES NO
16. Can you usually let yourself go and enjoy yourself at a lively party? .... YES NO
17. Are you an irritable person? .... YES NO
18. Should people always respect the law? .... YES NO
19. Have you ever blamed someone for doing something you knew was really your fault? .... YES NO
20. Do you enjoy meeting new people? .... YES NO
21. Are good manners very important? .... YES NO
22. Are your feelings easily hurt? .... YES NO
23. Are *all* your habits good and desirable ones? .... YES NO
24. Do you tend to keep in the background on social occasions? .... YES NO
25. Would you take drugs which may have strange or dangerous effects? .... YES NO
26. Do you often feel 'fed-up'? .... YES NO
27. Have you ever taken anything (even a pin or button) that belonged to someone else? .... YES NO
28. Do you like going out a lot? .... YES NO
29. Do you prefer to go your own way rather than act by the rules? .... YES NO
30. Do you enjoy hurting people you love? .... YES NO
31. Are you often troubled about feelings of guilt? .... YES NO
32. Do you sometimes talk about things you know nothing about? .... YES NO

continued

33. Do you prefer reading to meeting people? . . . YES NO
34. Do you have enemies who want to harm you? . . . YES NO
35. Would you call yourself a nervous person? . . . YES NO
36. Do you have many friends? . . . YES NO
37. Do you enjoy practical jokes that can sometimes really hurt people? . . . YES NO
38. Are you a worrier? . . . YES NO
39. As a child did you do as you were told immediately and without grumbling? . . . YES NO
40. Would you call yourself happy-go-lucky? . . . YES NO
41. Do good manners and cleanliness matter much to you? . . . YES NO
42. Have you often gone against your parents' wishes? . . . YES NO
43. Do you worry about awful things that might happen? . . . YES NO
44. Have you ever broken or lost something belonging to someone else? . . . YES NO
45. Do you usually take the initiative in making new friends? . . . YES NO
46. Would you call yourself tense or 'highly-strung'? . . . YES NO
47. Are you mostly quiet when you are with other people? . . . YES NO
48. Do you think marriage is old-fashioned and should be done away with? . . . YES NO
49. Do you sometimes boast a little? . . . YES NO
50. Are you more easy-going about right and wrong than most people? . . . YES NO
51. Can you easily get some life into a rather dull party? . . . YES NO
52. Do you worry about your health? . . . YES NO
53. Have you ever said anything bad or nasty about anyone? . . . YES NO
54. Do you enjoy co-operating with others? . . . YES NO
55. Do you like telling jokes and funny stories to your friends? . . . YES NO
56. Do most things taste the same to you? . . . YES NO
57. As a child were you ever cheeky to your parents? . . . YES NO
58. Do you like mixing with people? . . . YES NO
59. Does it worry you if you know there are mistakes in your work? . . . YES NO
60. Do you suffer from sleeplessness? . . . YES NO
61. Have people said that you sometimes act too rashly? . . . YES NO
62. Do you always wash before a meal? . . . YES NO
63. Do you nearly always have a 'ready answer' when people talk to you? . . . YES NO
64. Do you like to arrive at appointments in plenty of time? . . . YES NO
65. Have you often felt listless and tired for no reason? . . . YES NO
66. Have you ever cheated at a game? . . . YES NO
67. Do you like doing things in which you have to act quickly? . . . YES NO
68. Is (or was) your mother a good woman? . . . YES NO
69. Do you often make decisions on the spur of the moment? . . . YES NO
70. Do you often feel life is very dull? . . . YES NO
71. Have you ever taken advantage of someone? . . . YES NO
72. Do you often take on more activities than you have time for? . . . YES NO
73. Are there several people who keep trying to avoid you? . . . YES NO
74. Do you worry a lot about your looks? . . . YES NO
75. Do you think people spend too much time safeguarding their future with savings and insurance? . . . YES NO
76. Have you ever wished that you were dead? . . . YES NO
77. Would you dodge paying taxes if you were sure you could never be found out? . . . YES NO
78. Can you get a party going? . . . YES NO
79. Do you try not to be rude to people? . . . YES NO
80. Do you worry too long after an embarrassing experience? . . . YES NO
81. Do you generally 'look before you leap'? . . . YES NO
82. Have you ever insisted on having your own way? . . . YES NO
83. Do you suffer from 'nerves'? . . . YES NO
84. Do you often feel lonely? . . . YES NO
85. Can you on the whole trust people to tell the truth? . . . YES NO
86. Do you always practice what you preach? . . . YES NO
87. Are you easily hurt when people find fault with you or the work you do? . . . YES NO
88. Is it better to follow society's rules than go your own way? . . . YES NO
89. Have you ever been late for an appointment or work? . . . YES NO
90. Do you like plenty of bustle and excitement around you? . . . YES NO
91. Would you like other people to be afraid of you? . . . YES NO
92. Are you sometimes bubbling over with energy and sometimes very sluggish? . . . YES NO
93. Do you sometimes put off until tomorrow what you ought to do today? . . . YES NO
94. Do other people think of you as being very lively? . . . YES NO
95. Do people tell you a lot of lies? . . . YES NO
96. Do you believe one has special duties to one's family? . . . YES NO
97. Are you touchy about some things? . . . YES NO
98. Are you always willing to admit it when you have made a mistake? . . . YES NO
99. Would you feel very sorry for an animal caught in a trap? . . . YES NO
100. When your temper rises, do you find it difficult to control? . . . YES NO

PLEASE CHECK THAT YOU HAVE ANSWERED ALL THE QUESTIONS

APPENDIX 2

Short-scale EPQ-R

Age . . . .   Sex . . . .

1.	Does your mood often go up and down?	YES	NO
2.	Do you take much notice of what people think?	YES	NO
3.	Are you a talkative person?	YES	NO
4.	If you say you will do something, do you always keep your promise no matter how inconvenient it might be?	YES	NO
5.	Do you ever feel 'just miserable' for no reason?	YES	NO
6.	Would being in debt worry you?	YES	NO
7.	Are you rather lively?	YES	NO
8.	Were you ever greedy by helping yourself to more than your share of anything?	YES	NO
9.	Are you an irritable person?	YES	NO
10.	Would you take drugs which may have strange or dangerous effects?	YES	NO
11.	Do you enjoy meeting new people?	YES	NO
12.	Have you ever blamed someone for doing something you knew was really your fault?	YES	NO
13.	Are your feelings easily hurt?	YES	NO
14.	Do you prefer to go your own way rather than act by the rules?	YES	NO
15.	Can you usually let yourself go and enjoy yourself at a lively party?	YES	NO
16.	Are *all* your habits good and desirable ones?	YES	NO
17.	Do you often feel 'fed-up'?	YES	NO
18.	Do good manners and cleanliness matter much to you?	YES	NO
19.	Do you usually take the initiative in making new friends?	YES	NO
20.	Have you ever taken anything (even a pin or button) that belonged to someone else?	YES	NO
21.	Would you call yourself a nervous person?	YES	NO
22.	Do you think marriage is old-fashioned and should be done away with?	YES	NO
23.	Can you easily get some life into a rather dull party?	YES	NO
24.	Have you ever broken or lost something belonging to someone else?	YES	NO
25.	Are you a worrier?	YES	NO
26.	Do you enjoy co-operating with others?	YES	NO
27.	Do you tend to keep in the background on social occasions?	YES	NO
28.	Does it worry you if you know there are mistakes in your work?	YES	NO
29.	Have you ever said anything bad or nasty about anyone?	YES	NO
30.	Would you call yourself tense or 'highly-strung'?	YES	NO
31.	Do you think people spend too much time safeguarding their future with savings and insurances?	YES	NO
32.	Do you like mixing with people?	YES	NO
33.	As a child were you ever cheeky to your parents?	YES	NO
34.	Do you worry too long after an embarrassing experience?	YES	NO
35.	Do you try not to be rude to people?	YES	NO
36.	Do you like plenty of bustle and excitement around you?	YES	NO
37.	Have you ever cheated at a game?	YES	NO
38.	Do you suffer from 'nerves'?	YES	NO
39.	Would you like other people to be afraid of you?	YES	NO
40.	Have you ever taken advantage of someone?	YES	NO
41.	Are you mostly quiet when you are with other people?	YES	NO
42.	Do you often feel lonely?	YES	NO
43.	Is it better to follow society's rules than go your own way?	YES	NO
44.	Do other people think of you as being very lively?	YES	NO
45.	Do you always practice what you preach?	YES	NO
46.	Are you often troubled about feelings of guilt?	YES	NO
47.	Do you sometimes put off until tomorrow what you ought to do today?	YES	NO
48.	Can you get a party going?	YES	NO
